# Cluster size determines internal structure of transcription factories in human cells

**DOI:** 10.7554/eLife.103955

**Published:** 2026-07-20

**Authors:** Massimiliano Semeraro, Giuseppe Negro, Giada Forte, Antonio Suma, Giuseppe Gonnella, Peter Cook, Davide Marenduzzo

**Affiliations:** 1 Dipartimento Interateneo di Fisica, Università degli Studi di Bari and INFN, Sezione di Bari Bari Italy; 2 SUPA School of Physics and Astronomy, University of Edinburgh Edinburgh United Kingdom; 3 Sir William Dunn School of Pathology, University of Oxford Oxford United Kingdom; Colorado State University United States; National Cancer Institute United States

**Keywords:** chromatin, transcription factors, transcription factories, polymer models, mixed and demixed factories, Human

## Abstract

Transcription is a fundamental cellular process and the first step of gene expression. In human cells, it depends on the binding to chromatin of various proteins, including RNA polymerases and numerous transcription factors (TFs). Observations indicate that these proteins tend to form macromolecular clusters, known as *transcription factories*, whose morphology and composition are still debated. While some microscopy experiments have revealed the presence of *specialised factories*, composed of similar TFs transcribing families of related genes, sequencing experiments suggest instead that mixed clusters may be prevalent, as a panoply of different TFs binds promiscuously to the same chromatin region. The mechanisms underlying the formation of specialised or mixed factories remain elusive. With the aim of finding such mechanisms, here we develop a chromatin polymer model mimicking the chromatin binding-unbinding dynamics of different types of complexes of TFs. Surprisingly, both specialised (i.e. demixed) and mixed clusters spontaneously emerge, and which of the two types forms depends mainly on cluster size. The mechanism promoting mixing is the presence of non-specific interactions between chromatin and proteins, which become increasingly important as clusters become larger. This result, that we observe both in simple polymer models and more realistic ones for human chromosomes, reconciles the apparently contrasting experimental results obtained. Additionally, we show how the introduction of different types of TFs strongly affects the emergence of transcriptional networks, providing a pathway to investigate transcriptional changes following gene editing or naturally occurring mutations.

## Introduction

The 3D organisation of chromatin, the filament composed of DNA wrapped around histone proteins which constitutes the building block of mammalian chromosomes, is a dynamic and intricate blueprint that is thought to be important for cellular function and gene expression (Chiang et al., 2022a). Recent advances in microscopy and high-throughput sequencing (Kempfer and Pombo, 2020) have revealed a rich hierarchy of 3D chromatin structures within the cell nucleus. These range from relatively small DNA loops of tens to hundreds of base pairs (bps), to large organised domains spanning over hundreds of thousands of base pairs (or kilobase pairs, kbp), which are referred to as topologically-associating domains (TADs), whose segments interact more frequently among each other than with other parts of the genome. At even larger scales, the genomic material divides into A (active) and B (inactive) compartments, which have different gene activity and 3D compaction, whereas different chromosomes occupy distinct territories inside the nucleus (Pombo and Dillon, 2015; Lieberman-Aiden et al., 2009; Dixon et al., 2012).

A central question in cellular biology is the extent to which this rich and multi-scale organisation is influenced, or even driven, by transcription (Cook and Marenduzzo, 2018), the fundamental biological process during which the information encoded in a segment of DNA is converted into RNA, to be then translated into proteins. On the one hand, it is widely believed that TADs remain largely invariant in cells with very different transcriptional programmes (e.g. belonging to different organs) – which points to little role for transcription in determining structure Dixon et al., 2016 (for an opposing view, see Rowley et al., 2017). On the other hand, enzymes engaged in the process of transcription, known as RNA polymerases, tend to form aggregates inside the nucleus, often referred to as phase-separated condensates, hubs, or transcription factories (Papantonis and Cook, 2013; Cook and Marenduzzo, 2018; Cramer, 2019; Brackley et al., 2021; Chiang et al., 2022b). Being attached to a factory strongly enhances the transcriptional activity of a gene (Papantonis and Cook, 2013; Cook and Marenduzzo, 2018), therefore factories are a primary example of a structural unit with a clear transcriptional role.

A recent effective way to investigate this intricate connection between transcription and 3D chromatin structure has been provided by polymer models together with Brownian dynamics simulations (Bianco et al., 2017; Laghmach et al., 2021; Di Pierro et al., 2018; Barbieri et al., 2012; Bianco et al., 2018; Jost and Vaillant, 2018; Ghosh and Jost, 2020; Lin et al., 2021; Natesan et al., 2022; Bianco et al., 2017; Chiariello et al., 2016; Giorgetti et al., 2014; Michieletto et al., 2016; Di Pierro et al., 2017). This in silico approach has pointed to a simple and generic mechanism – the bridging-induced attraction or bridging induced phase separation – that spontaneously drives formation of transcription factories (Brackley et al., 2016; Brackley et al., 2013). Such microphase separation is due to the fact that, in this type of modelling, TF and polymerase complexes (TF:pol) are usually depicted as multivalent elements, so that each of them can simultaneously bind to several chromatin sites: this is reasonable as a complex of proteins can easily have more than one chromatin-binding domain. Multivalent binding triggers a positive feedback: a TF:pol binding to the chromatin filament provokes a local increase of chromatin density as it attracts several chromatin sites. The higher chromatin density, in turn, recruits further TF:pol resulting in a cluster, or transcription factory, formation. This feedback is eventually arrested by entropic costs associated with crowding and looping more and more DNA (Marenduzzo and Orlandini, 2009; Brackley et al., 2016).

The existence of clusters prompts the question: does a typical cluster mainly contain just one kind of TF, or many different ones? On the one hand, microscopy experiments suggest that different factories specialise in transcribing different sets of genes, so that any one factory typically contains mainly one type of TF. For example, active forms of RNA polymerases II and III are each housed in distinct nucleoplasmic factories that make genic and snRNA transcripts, respectively (Albert and Kruglyak, 2015; Cook, 2001; Papantonis and Cook, 2013). Similarly, distinct ERα, KLF1, and NFκB factories specialise in transcribing genes involved in the estrogen response, globin production, and inflammation (Fullwood et al., 2009; Schoenfelder et al., 2010; Papantonis et al., 2012). One important consequence of the formation of such specialised factories is the creation of 3D networks (Pancaldi et al., 2016), in which genes sharing the same TFs are co-transcribed in the same clusters.

On the other hand, and in contrast to the evidence for specialised factories, chromatin immuno-precipitation (ChIP) techniques have revealed the existence of particular chromatin fragments, called ‘highly-occupied targets’ (or HOT), which are promiscuously bound by several different TFs (Moorman et al., 2006; Foley and Sidow, 2013; Cortini and Filion, 2018). Additionally, single-cell transcriptional profiling points to expression levels varying continuously as cells differentiate into other cell types, which points to a complex interplay between many factors, rather than a few acting as binary switches (Ding et al., 2022; Elmentaite et al., 2022). Interestingly, simulations of the types described above which involve two different kinds of TFs, each one binding specifically to two different TU types, show the resulting clusters often contain bound TFs of just one type, rather than mixtures, although this aspect has not been investigated in depth (Brackley et al., 2013; Brackey et al., 2020; Brackley et al., 2021; Buckle et al., 2018; Bianco et al., 2017; Brackley et al., 2017a; Brackley et al., 2016; Nicodemi and Pombo, 2014; Conte et al., 2022; Semeraro et al., 2023b; Tiana et al., 2016; Giorgetti et al., 2014; Crippa et al., 2020; Semeraro et al., 2023b; [Bibr bib68][Bibr bib85]).

Here, we develop a polymer model with the aim of investigating the mechanisms leading to the formation of specialised or mixed factories: i.e., clusters formed by a single type or by multiple types of TFs, respectively. Within our framework, chromatin is depicted as a polymer composed of a multicolour sequence of beads, corresponding to transcription units (TUs) of different types (or colours), each one binding to the corresponding type of transcription factors, represented as additional spheres diffusing in the system. With respect to previous works on multicolour models (Brackley et al., 2016; Bianco et al., 2018; Chiariello et al., 2016; Jost et al., 2014; Falk et al., 2019; Johnstone et al., 2020), there are two important differences. First, here we model chromatin transcription by making the assumption that a chromatin bead is transcribed when it binds to a TF. In this way we are able to investigate the link between 3D structure of active chromatin and transcription (transcriptional patterns and emerging transcriptional correlation networks), rather than solely structure as done in previous models. Second, we study the morphology of the ensuing transcriptional clusters, studying their composition and the way in which it can be affected by the 1D binding landscape, and the balance between non-specific and specific chromatin-TF interactions.

Our main result is that specialised (demixed) and mixed clusters are *not* mutually exclusive. More specifically, we unexpectedly find a transition, or crossover, between a specialised and a mixed clusters regime, influenced by the size of emerging clusters. Smaller clusters are typically specialised, whereas larger clusters are more likely to be formed by different TF types. This result enables us to reconcile the apparently contrasting experimental observations cited above: it is no longer surprising that specialised and mixed clusters can coexist within the same cell, as cluster size depends, for instance, on the local 1D pattern of TU binding sites along chromatin. It is also important to specify that, even if an investigation of a transition between small and larger transcription factories has been carried out in mouse embryonic stem cells (Cho et al., 2018), in serum-stimulated cells (Wei et al., 2020) and in zebrafish embryos (Pancholi et al., 2021), our study aims to provide a broader mechanistic overview. We further integrated our multi-colour model with experimental data, specifically DNase hypersensitive site (DHS) locations, to study human chromosomes. Here, two colours are considered as the simplest extension of the previous DHS model with one colour (Brackley et al., 2021), by distinguishing between active TF:pol complex that bind, respectively, to cell-type-invariant and cell-type-specific TUs in strings mimicking whole human chromosomes. Finally, the existence of specialised and mixed factories is further validated by incorporating different types of proteins into more complex and realistic chromatin models, such as the ‘highly predictive heteromorphic polymer model’ (HiP-HoP model), which accounts for loop extrusion by cohesin-like complexes, the presence of inactive or silenced chromatin, and chromatin heteromorphism (Buckle et al., 2018).

## Results

### Toy model with different transcription factors

**Figure 1.**
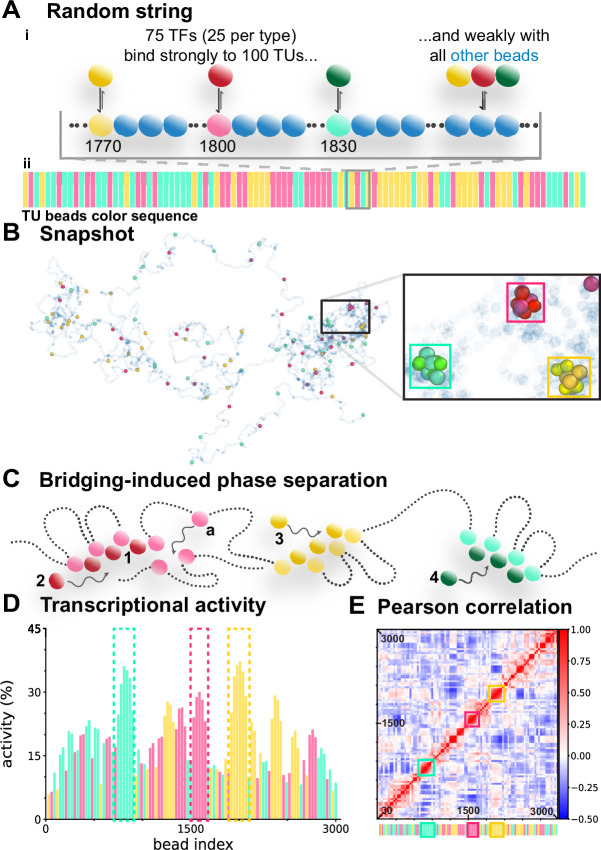
Toy model, with transcription units (TUs) coloured randomly (the random string). (**A**) Overview. (**i**) Yellow, red, and green TFs (25 of each colour) bind strongly (when in an on state) to 100 TUs beads of the same colour in a string of 300 beads (representing 3 Mb), and weakly to blue beads. TU beads are positioned regularly and coloured randomly. TFs switch between off and on states at rates $\alpha_{off}=10^{-5} \tau_{B}^{-1}$ and $\alpha_{on}=\alpha_{off/4}$ ($\tau_{B}$ Brownian time, see SI). (ii) The sequence of bars reflects the random sequence of yellow, red, and green TUs (blue beads not shown). (**B**) Snapshot of a typical conformation obtained after a simulation (transcription factors, TFs not shown). Inset: enlargement of boxed area. TU beads of the same colour tend to cluster and organise blue beads into loops. (**C**) Bridging-induced phase separation drives clustering and looping. Local concentrations of red, yellow, and green TUs and TFs might appear early during the simulation. Red TF 1 – which is multivalent – has bound to two red TUs and so forms a molecular bridge that stabilises a loop; when it dissociates it is likely to re-bind to one of the nearby red TUs. As red TU 2 diffuses through the local concentration, it is also likely to be caught. Consequently, positive feedback drives growth of the red cluster (until limited by molecular crowding). Similarly, the yellow and green clusters grow as yellow TF 3 and green TF 4 are captured. (**D**) Bar heights give transcriptional activities of each TU in the string (average of 100 runs each lasting $8\, 10^{5}\tau_{B}$). A TU bead is considered to be active whilst within $2.24\sigma\sim 6.7\times 10^{-8}m$ of a TF:pol complex of similar colour. Dashed boxes: regions giving the three clusters in the inset in (**B**). (**E**) Pearson correlation matrix for the activity of all TUs in the string.

To try to solve the apparently contrasting views of segregated and mixed factories we start by introducing a new simple polymer model, where a 9 Mbp chromatin fragment is represented by a chain of 3000 beads (each 30 nm in diameter, and corresponding to 3 kbp). Different kinds of TU beads are positioned regularly in this string, but are coloured randomly yellow, red, or green (we refer to this case as the random string). Different kinds of TFs are modelled as diffusing spheres, at first approximation of the same size of chromatin beads (see later for simulations changing the TFs size), which bind reversibly and strongly to beads of the same color, and weakly to all others (see Figure 1A, and Methods for further details). Figure 1B shows a typical 3D conformation found in the steady state after running a Brownian-dynamics simulation. Remarkably, clusters of TUs and TFs with distinct colours appear and disappear spontaneously. Such clustering is driven by the positive feedback illustrated in Figure 1C; it depends critically on TFs being able to form molecular bridges that anchor loops (Brackley et al., 2021; Brackley et al., 2016; Brackley et al., 2013).

**Figure 1—figure supplement 1.**
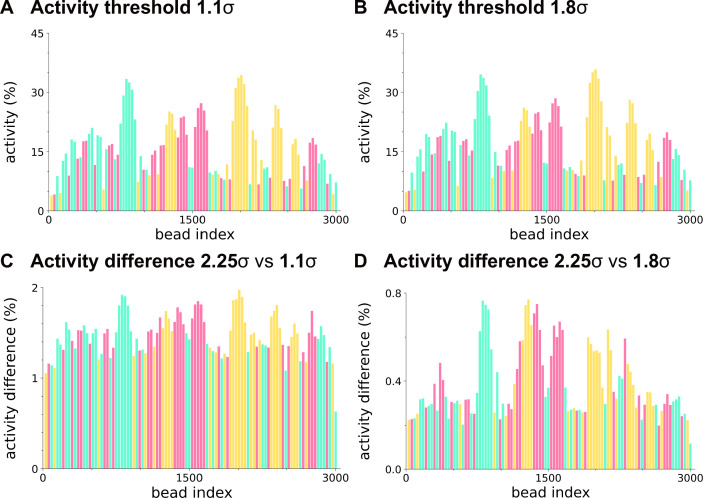
Varying transcriptional activity threshold does not alter transcriptional profiles. (**A** and **B**) Bar heights give transcriptional activities of each transcription unit (TU) in the toy string (average of 100 runs each lasting $8\,\times 10^{5}\tau_{B}$). A TU bead is considered to be active, respectively, whilst within $1.8\sigma\sim 5.4\times 10^{-8}m$ and $1.1\sigma\sim 3.3\times 10^{-8}m$ of a TF:pol complex of similar colour. (**C** and **D**) Difference between the transcriptional profile obtained with threshold $2.25\sigma$ (see Figure 1D) and those obtained with threshold $1.8\sigma$ and, $1.1\sigma$, respectively.

**Figure 1—figure supplement 2.**
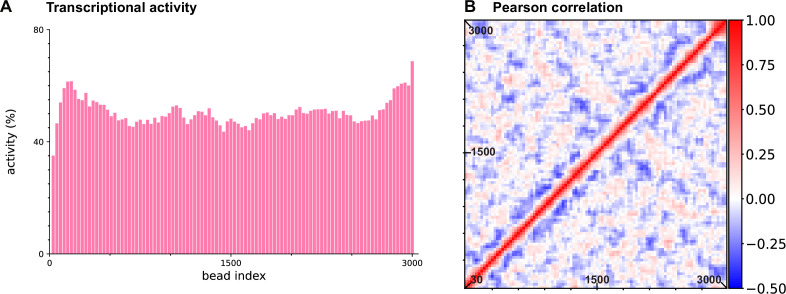
Random string with just one colour. Transcription units (TUs) in the random string considered in the main text are coloured randomly yellow, red, or green; here, instead, every TU has the same colour. Averages are obtained from 100 runs, each lasting $8\,\times 10^{5}\tau_{B}$, (**A**) The transcriptional activity profile is much flatter than that obtained with the 3-colour version. (**B**) The Pearson correlation matrix also lacks red blocks along the diagonal.

We now assume that the spheres represent TF:pol complexes, and make the reasonable assumption that a TU bead is transcribed if it lies within 2.25 diameters ($2.25\sigma$) of a complex of the same colour (small changes in this threshold do not affect qualitatively the measured activity, as shown in Figure 1—figure supplement 1); then, the transcriptional activity of each TU is given by the fraction of time that the TU and a TF:pol lie close together. Figure 1D reports the mean activity profile down the string obtained combining transcriptional data from 100 independent simulation runs each lasting $8\, 10^{5}\tau_{B}$; TUs with the lowest activities are flanked by differently-coloured TUs, while those with the highest activities are flanked by similarly-coloured TUs (dashed rectangles in Figure 1D). As expected, a single-colour model with the same TU placement leads to a flat activity profile (Figure 1—figure supplement 2). Clearly, close proximity in 1D genomic space favours formation of similarly-coloured clusters.

We next examine how closely transcriptional activities of different TUs correlate (Cohen et al., 2000); the Pearson correlation matrix for all TUs is shown in Figure 1E. This is built in the following way: for every couple $i-j$ of TUs, we evaluate the Pearson correlation coefficient between the transcription of $i$ and $j$, and then we colour the $ij$ (and $ji$) pixel of the matrix accordingly. Correlations between neighbouring TUs of similar colour are often positive and strong, resulting in square red blocks along the diagonal (coloured boxes in Figure 1E highlight the three clusters shown in the zoom in Figure 1B). This effect is again due to the self-assembly of clusters containing neighbouring TUs of the same colour. In contrast, neighbours with different colours tend to compete with each other for TF:pols, and so down-regulate each other to yield smaller correlations. Correlations are more trivial in the single-colour counterpart of Figure 1, where the matrix yields only a positive-correlation band along the diagonal (Figure 1—figure supplement 2). These results provide simple explanations of two mysterious effects – the first being why adjacent TUs throughout large domains tend to be co-transcribed so frequently (Gilbert et al., 2004). The second concerns how expression quantitative trait loci (eQTLs)—genomic regions that are statistically associated with variation in gene expression levels—function. While current models often attribute their effects to post-transcriptional regulation through complex mechanisms (Boyle et al., 2017; Brackley et al., 2021), we have previously argued that any transcriptional unit (TU) can act as an eQTL by directly influencing gene expression at the transcriptional level (Brackley et al., 2021). Here, we observe individual TUs up-regulating or down-regulating the activity of other TUs – hallmark behaviours of eQTLs that can give rise to genetic effects such as ‘transgressive segregation’ (Brem and Kruglyak, 2005). This phenomenon refers to cases in which alleles exhibit significantly higher or lower expression of a target gene, and can be, for instance, caused by the creation of a non-parental allele with a specific combination of QTLs with opposing effects on the target gene.

### Local mutations

**Figure 2.**
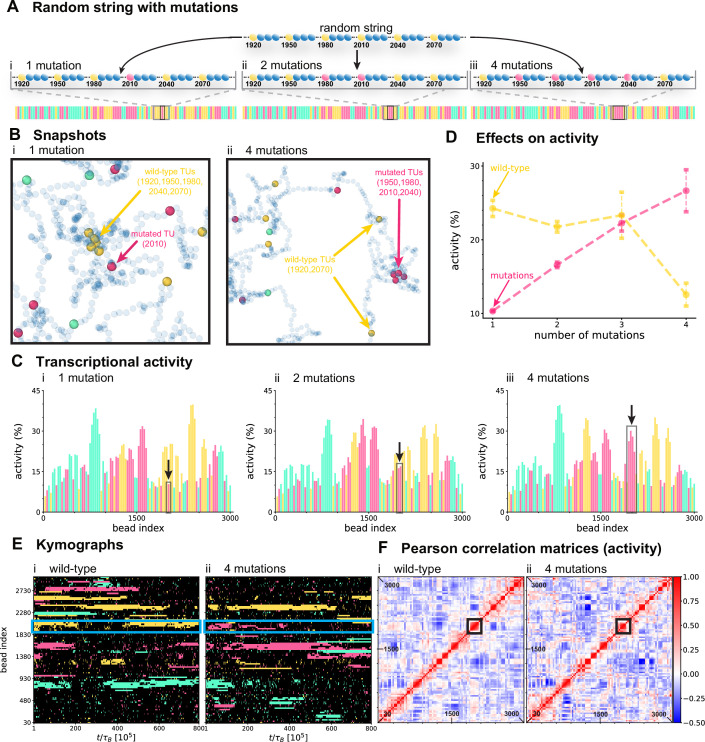
Simulating effects of mutations. Yellow transcription unit (TU) beads 1920, 1950, 1980, 2010, 2040, and 2070 in the random string have the highest transcriptional activity. One to four of these beads are now mutated by recolouring them red. (**A**) The sequence of bars reflects the sequence of yellow, red, and green TUs in random strings with 1, 2, and 4 mutations (blue beads not shown). Black boxes highlight mutant locations. (**B**) Typical snapshots of conformations with (**i**) one, and (ii) four mutations. (**C**) Transcriptional-activity profiles of mutants (averages over 100 runs, each lasting $8\,\times 10^{5}\tau_{B}$). Bars are coloured according to TU colour. Black boxes: activities of mutated TUs. (**D**) Activities (+/- SDs) of wild-type (yellow) and different mutants. Three mutations: TUs 1950, 1980, and 2010 mutated from yellow to red. (**E**) Typical kymographs for (**i**) wild-type, corresponding to the same original string presented in Figure 1, and (ii) 4-mutant cases, in which four yellow TUs have been mutated to red. Each row reports the transcriptional state of a TU during one simulation. Black pixels denote inactivity, and others activity; pixels colour reflects TU colour. Blue boxes: region containing mutations. (**F**) Pearson correlation matrices for wild-type and 4-mutant cases. Black boxes: regions containing mutations (mutations also change patterns far from boxes).

To explore the impact of introducing different colours, we characterise the effects of local mutations. We choose the most active region in the random string – one containing a succession of yellow TUs – and ‘mutate’ 1 to 4 of these TUs by recolouring them red (Figure 2A). These simulations are inspired by editing experiments performed using CRISPR/Cas9 (Morgan et al., 2017). Typical snapshots show red mutants are often ejected from yellow clusters (Figure 2Bi), or cluster together to leave their wild-type neighbours in isolation (Figure 2Bii). These changes are reflected in activity profiles (Figure 2C; arrows indicate mutations). As the number of mutations in the cluster increase, activities of yellow beads in that cluster decrease (Figure 2D), and new red clusters often emerge (Figure 2B and ii; Figure 2Ciii).

To confirm that four mutations in a yellow cluster often lead to the development of a red cluster, we monitor cluster dynamics over time. Figure 2Ei illustrates a typical kymograph illustrating changes in activity of all TUs in the wild-type, corresponding to the same original string presented in Figure 1; yellow, red, and green pixels mark activity of respective TUs, and black ones inactivity. In this particular simulation, a yellow cluster in the region that will be mutated (marked by the blue rectangle) is present during the first quarter of the time window; it then disappears to reappear half-way through the window and then persists until the end. From the activity profiles in Figure 2C, we can observe that as the number of mutations increases, the yellow cluster is replaced by a red cluster, with the remaining yellow TUs in the region being expelled (Figure 2Bii). This behaviour is reflected in the dynamics, as seen by comparing panels Ei and Eii: in the string with four mutations, transcription of the yellow TUs is inhibited in the affected region, while prominent red stripes—corresponding to active, transcribing clusters—emerge (Figure 2Eii). Pearson correlation matrices provide complementary information: the yellow cluster in the wild-type yields a solid red block indicating strong positive correlations (Figure 2Fi), while most of the pixels of this block become light-red or white in the string with 4 mutations (Figure 2Fii). These results confirm that local arrangements of TUs on the genetic map determine the extent to which any particular TU will cluster and so become active.

### Variations in TF concentration

**Figure 3.**
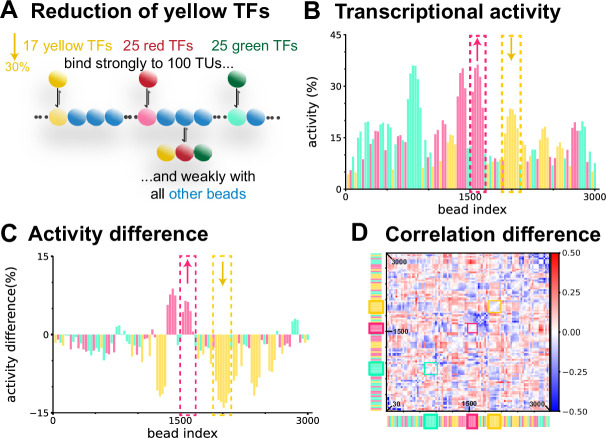
Reducing the concentration of yellow transcription factors (TFs) reduces the transcriptional activity of most yellow transcription units (TUs) while enhancing the activities of some red TUs. (**A**) Overview. Simulations are run using the random string with the concentration of yellow TFs reduced by 30%, and activities determined (means from 100 runs each lasting $8\, \times 10^{5}\tau_{B}$). (**B**) Activity profile. Dashed boxes: activities fall in the region containing the biggest cluster of yellow TUs seen with 100% TFs, as those of an adjacent red cluster increase. (**C**) Differences in activity induced by reducing the concentration of yellow TFs. This plot is obtained by subtracting the transcriptional activity of the wild-type, Figure 1D, from that of the current system in panel B. (**D**) Pearson correlation difference matrix. This plot is obtained by subtracting the Pearson correlation matrix of the wild-type, Figure 1E, from that of the current system. Boxes: regions giving the 3three clusters from Figure 1B, inset.

The concentration of TFs is expected to influence the global activity patterns observed and can be adjusted in our model accordingly. These simulations are motivated by experiments that reduce global TF levels using auxin-induced degrons (Luan et al., 2021). Specifically, we reduce the concentration of yellow TFs binding to the random string by 30% (Figure 3A). As expected, transcriptional activity falls both globally and locally (see yellow dotted rectangles in Figure 3B and C). Surprisingly, activity of a nearby cluster of red TUs (numbers 1080, 1110, 1170, 1200, and 1530 to 1650) increases by 50% (red dotted rectangles in Figure 3B and C). This effect is specific, in the sense that there is little effect on green clusters (e.g. compare Figure 1D with Figure 3B). We attribute this to a now-reduced steric competition for 3D space by yellow neighbours – fewer yellow clusters are present to stunt growth of nearby red ones.

Figure 3D shows the difference in correlation between the case with reduced yellow TFs and the case displayed in Figure 1E. We can notice a change in correlation between the yellow cluster (boxed) and its neighbour clusters, even if distant. For instance, yellow clusters are more probable to be found both turned off due to the lack of yellow TFs, and thus their activation becomes more correlated. At the same time, when yellow clusters are turned off the activation of other clusters can be affected, with an increase or decrease of correlation. Overall, these results reveal numerous statistically significant correlations in gene activity both in proximal and distal regions of the genetic map. This observation aligns with the omnigenic model, which proposes that the manifestation of a genetic trait is influenced not only by the expression of core genes directly associated with the trait, but also by peripheral genes, which can exert indirect effects through gene regulatory networks (Boyle et al., 2017).

### Effects of 1D TU patterns on transcriptional activity

**Figure 4.**
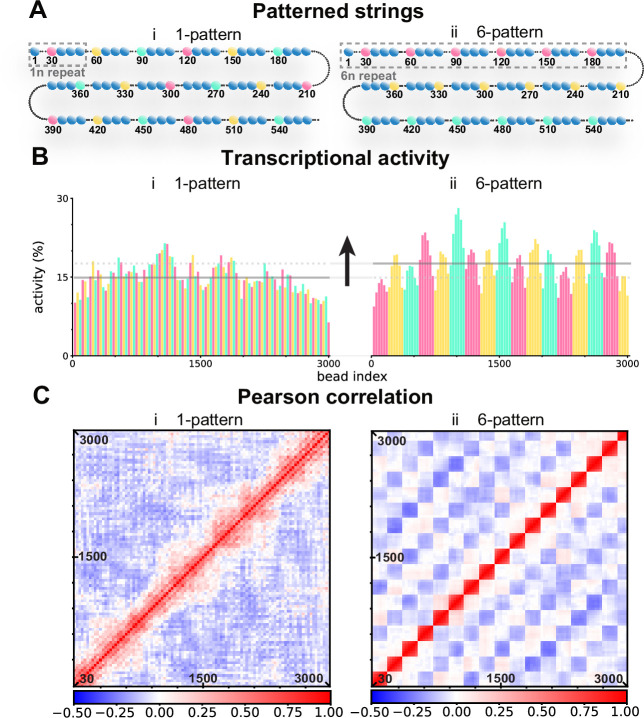
Clustering similar transcription units (TUs) in 1D genomic space increases transcriptional activity. (**A**) Simulations involve toy strings with patterns (dashed boxes) repeated one or six times. Activity profiles plus Pearson correlation matrices are determined (100 runs, each lasting $8\,\times 10^{5}\tau_{B}$). (**B**) The 6-pattern yields a higher mean transcriptional activity (arrow highlights difference between the two means). (**C**) The 6-pattern yields higher positive correlations between TUs within each pattern and higher negative correlations between each repeat.

To gain a deeper understanding of the local effects revealed by the random string, we now analyse and compare various toy strings that feature regular and repeating patterns of coloured TUs (Figure 4 and methods for further details). Two results are apparent. First, activities (Figure 4Bii) in the 6-pattern case are higher overall (compare horizontal dotted lines), and more variable (compare activities of the two central TUs within each repeat with peripheral ones) relative to the 1-pattern case (Figure 4Bi). This is consistent with positive additive effects acting centrally within each 6-pattern repeat, coupled to competitive negative effects of flanking and differently-coloured repeats at the edges. Second, the 6-pattern also has a Pearson correlation matrix (Figure 4Cii) that is highly-structured, with a checkerboard pattern; red blocks on the diagonal indicate high positive correlations (so the 1D 6-pattern clearly favours 3D clustering). [Such a checkerboard pattern is not seen with a single-colour model that has a correlation matrix with one red continuous diagonal when TUs are regularly spaced Figure 1—figure supplement 2.] Additionally, blue off-diagonal blocks indicate repeating negative correlations that reflect the period of the 6-pattern. These results show how strongly TU position in 1D genomic space affects 3D clustering and activity, and that these effects depend on inclusion of more than one colour.

### Emergent transcriptional correlation networks

**Figure 5.**
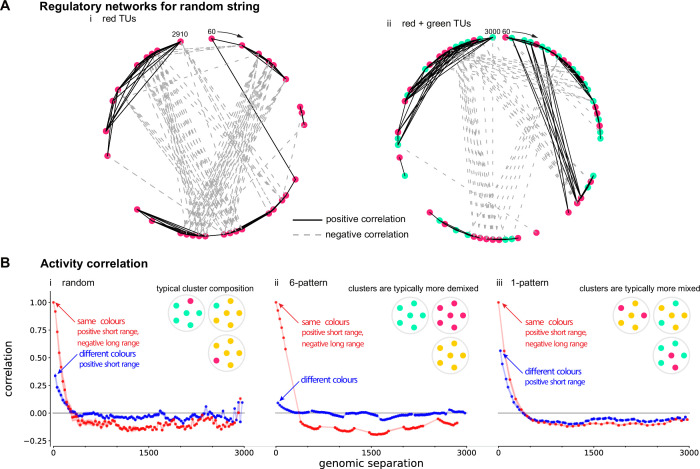
Transcription unit (TU) transcriptional networks and demixing. Simulations are run using the toy models indicated, and complete correlation networks (qualitatively reminiscent of gene regulatory networks) constructed from Pearson correlation matrices. (**A**) Simplified network given by the random string. TUs from first (bead 30) to last (bead 3000) are shown as peripheral nodes (coloured according to TU); black and dashed grey edges denote statistically-significant positive and negative correlations, respectively (above a threshold of 0.2, corresponding to a *p*-value $\sim 5\, \times 10^{-2}$). The complete network consists of $n=100$ individual TUs, so that there are $n_{c}=\binom{100}{2}=4950$ pairs of TUs couples; we find 990 black and 742 gray edges. Since *p*-value.$ n_{c}=223$, most interactions (edges) are statistically significant. Networks shown here only correlations (**i**) between red TUs, and (ii) between red and green TUs. (ii) (**B**) Average Pearson correlation (shading shows +/- SD, and is usually less than line/spot thickness) as a function of genomic separation for the (**i**) random, (ii) 6-, and (iii) 1-pattern cases. Correlation values at fixed genomic distance are taken from super-/sub-diagonals of Pearson matrices. Red dots give mean correlation between TUs of the same colour (three possible combinations), and blue dots those between TUs of different colours (4four possible combinations). Cartoons depict contents of typical clusters to give a pictorial representation of mixing degree (as this determines correlation patterns); see SI for exact values of $\theta_{\rm dem}$.

We have seen many positive and negative correlations between activities of TUs in the random string (Figure 1). We now select significant correlations from Pearson correlation matrices (those which are >0.2, Figure 5A) to highlight emergent interaction networks (Brackley et al., 2021). In such networks, nodes represent each TU from first to last (other beads are not shown), and edges indicate positive (black) or negative (grey) correlations in activities of node pairs. Even for the toy random string, these networks prove to be very complex (Figure 5—figure supplement 1). They are also ‘small-world’ (i.e. most nodes can be reached from other ones by a few steps Watts and Strogatz, 1998; Brackley et al., 2021). Given this complexity, we now consider simplified versions. Thus, in Figure 5Ai, only interactions between red TUs are shown (the first red TU is at position 60, the last at position 2910, and interactions between different colours are not depicted). As expected, activities of most red TUs are positively correlated with those of nearby TUs. Conversely, negative correlations connect distant TUs, as found in the single-colour model (Brackley et al., 2021); as we have seen, binding of red TFs to any red cluster reduces the number available to bind elsewhere.

**Figure 5—figure supplement 1.**
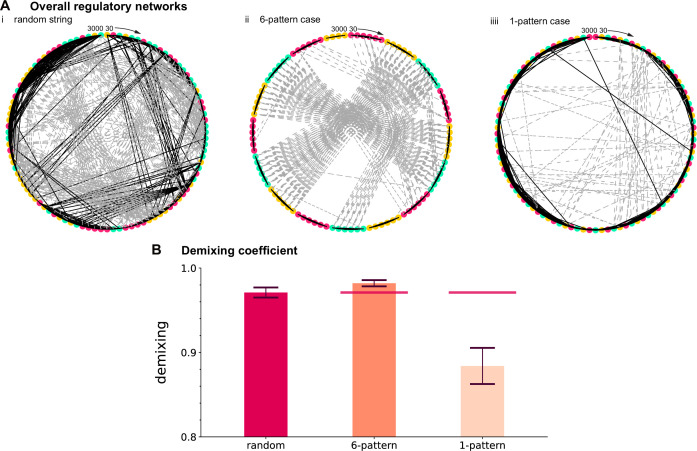
Complete regulatory networks, and demixing coefficients for toy strings. (**A**) Networks involving positive (black) and negative (dashed grey) correlations between all coloured beads are presented, for the models considered in the main text in Figure 5. (**i**) The random string. The network is complex and entails both positive and negative correlations. (**ii**) The 6-pattern repeat. There are many black edges within repeats and virtually none between repeats, many grey edges between repeats, and few edges between transcription units (TUs) with different colours. (**iii**) The 1-pattern repeat. There are few long black edges. (**B**) Average of $\theta_{\rm dem}$ (error bars: weighted SD) for various toy strings. Red lines: values for random string. Compared to the random case, 6-pattern is more demixed, and the 1-pattern less demixed. As discussed in the main text, same-colour and different-colour correlations diverge from each other in the 6-pattern case, and roughly overlap in the 1-pattern.

**Figure 5—figure supplement 2.**
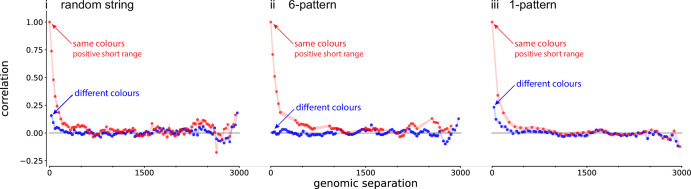
Activity correlations found in toy chromosomes without weakly-binding beads. Correlation values (shading shows +/- SD, and is usually less than line/spot thickness) at fixed genomic distance are taken from super-/sub-diagonals of Pearson matrices. Red dots give mean correlations between transcription units (TUs) of same colour, and blue dots those between TUs of different colours. (**i**) Random string, (**ii**) 6-pattern string, and (**iii**) 1-pattern string with non-binding beads instead of weakly-binding ones.

In Figure 5Aii, we consider just interactions between red TUs and green TUs. Remarkably, close-range positive correlations (black edges) are still seen between TU pairs that no longer bind TUs of the same colour. We suggest this is due to the presence of weakly-binding beads. Specifically, a red cluster organises a surrounding cloud of weakly-binding beads, and these will bind some green TFs that – in turn – bind green TUs. In contrast to the same-colour network in Figure 5Ai, there are now more long-range positive correlations, showing that the presence of multiple colours enriches the emerging network.

To obtain further quantitative insight into these subtle yet remarkable correlations, we compute the average of those between same- and different-colour TUs as a function of genomic separation (Figure 5B). For the random string, same-colour correlations switch from clearly positive to slightly negative at about 300 beads (Figure 5Bi, red curve). Differently-coloured correlations yield a broadly-similar switch, although positive and negative values are weaker (Figure 5Bi, blue curve). The 6-pattern gives qualitatively similar trends, with the magnitude of differently-coloured correlations dampened further (Figure 5Bii). In contrast, the 1-pattern string yields largely overlapping curves (Figure 5Biii). These results illustrate how the sequence of TUs on a string can strikingly affect formation of mixed clusters; they also provide an explanation of why activities of human TUs within genomic regions of hundreds of kbp are positively correlated (Hurst et al., 2004).

To quantify the extent to which TFs of different colours share clusters, we introduce a demixing coefficient, $\theta_{\rm dem}$ (see Methods for definition), which can vary between 0 and 1. If $\theta_{\rm dem}=1$, a cluster contains only TFs of one colour (and so is fully demixed); if $\theta_{\rm dem}=0$, it contains both red and green TFs in equal numbers (and so is fully mixed). Intuitively, one might expect $\theta_{\rm dem}$ to fall as the number of adjacent TUs of similar colour in a string fall; this is what is seen with the 6- and 1-patterns – strings with the most and least numbers of adjacent TUs of similar colour, respectively (Figure 5—figure supplement 1; shown schematically by the cluster cartoons in Figure 5B).

Our simulations then show that in cases where same- and different-colour Pearson correlations trends overlap (as in the 1-pattern string, see Figure 5Biii), clusters are more mixed (have a larger value of $\theta_{\rm dem}$). Instead, in cases where same- and different-colour correlations trends do not overlap, or are more different (as in the 6-pattern string, see Figure 5Bii), then clusters are typically unmixed, and so have a larger value of $\theta_{\rm dem}$ (Figure 5—figure supplement 1). These results on activity correlation and TF cluster composition suggest that, if eQTLs act transcriptionally as expected (Brackley et al., 2021), down-regulating eQTLs are likely to be located further from their target genes than up-regulating ones. In addition, it is important to note that mixing is promoted by the presence of weakly binding beads; replacing these with non-interacting ones leads to increased demixing and a reduction in long-range negative correlations (Figure 5—figure supplement 2). More generally, our findings indicate that the presence of multiple TF colours offers an effective mechanism to enrich and fine-tune transcriptional regulation.

### Transcriptional activity and comparison with real human chromosomes

**Figure 6.**
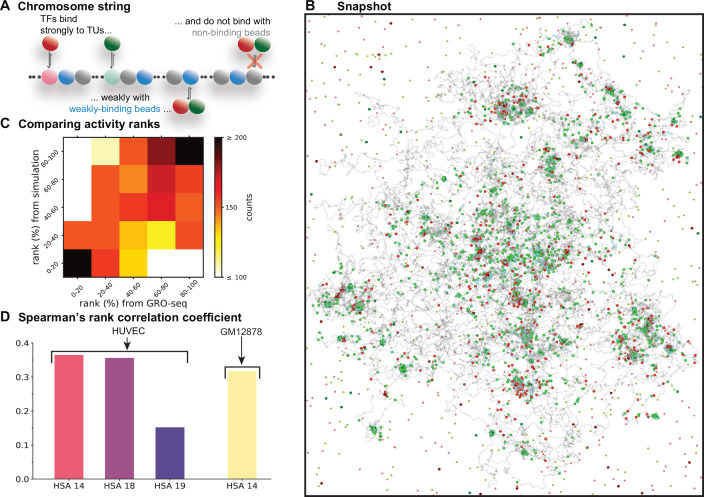
Comparison of transcriptional activities of transcription units (TUs) on different human chromosomes determined from simulations and GRO-seq. (**A**) Overview of panels (**A–C**). The 35784 beads on a string representing HSA14 in HUVECs are of four types: TUs active only in HUVECs (red), ‘house-keeping’ TUs – ones active in both HUVECs and ESCs (green), ‘euchromatic’ ones (blue), and ‘heterochromatic’ ones (gray). Red and green transcription factors (TFs) bind to TUs of the same colour with strong (specific) interactions, while they experience a weak (non-specific) attraction to euchromatin. Interactions between both red and green TFs and heterochromatin are purely repulsive. (**B**) Snapshot of a typical conformation, showing both specialised and mixed clusters. (**C**) TU activities seen in simulations and GRO-seq are ranked from high to low, binned into quintiles, and activities compared. (**D**) Spearman’s rank correlation coefficients for the comparison between activity data obtained from analogous simulations and GRO-seq for the chromosomes and cell types indicated.

We now simulate human chromosome 14 (HSA 14) in HUVECs, with individual beads in the string coloured appropriately (Figure 6A). Thus, TUs transcribed uniquely in HUVECs are coloured red, housekeeping TUs (i.e. ones also expressed in a stem cell, namely H1-hESCs) are green, euchromatic regions blue, and heterochromatic ones gray. Figure 6B shows a typical snapshot; red and green clusters again form spontaneously. We next determine transcriptional activities, rank them in order from high to low, and compare binned rank orders with those obtained experimentally by GRO-seq (Figure 6C); most counts lie along the diagonal, meaning there is a good agreement between the two data sets. More quantitatively, Spearman’s rank correlation coefficient is 3.66×10^−1^, which compares with 3.24×10^−1^ obtained previously using a single-colour model (Brackley et al., 2021). In both cases the estimated uncertainty is of order 10^−3^ (mean and SD obtained using the bootstrap technique over 100 trials) and the *p*-value is <10^−6^ (2-sided t-test), showing statistical significance.

**Figure 6—figure supplement 1.**
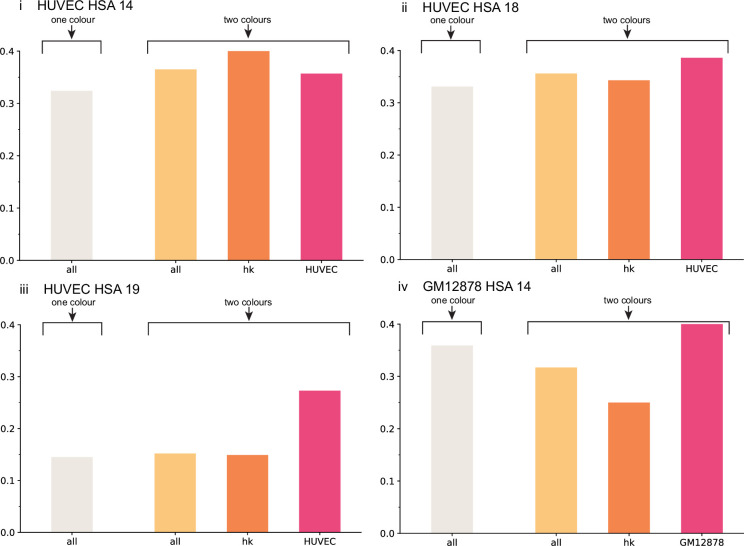
Spearman’s rank-correlation coefficients determined by comparing activity data obtained from simulations (one- and two-colour) and GRO-seq for chromosomes and cell types indicated. Grey bars: one-colour cases. Coloured bars: two-colour cases where values are for ‘all’ TUs, just housekeeping (‘hk’), and HUVEC- or GM12878-specific ones. (**i**) HSA 14 in HUVEC. (**ii**) HSA 18 in HUVEC. (**iii**) HSA 19 in HUVEC. (**iv**) HSA 14 in GM12878.

**Figure 6—figure supplement 2.**
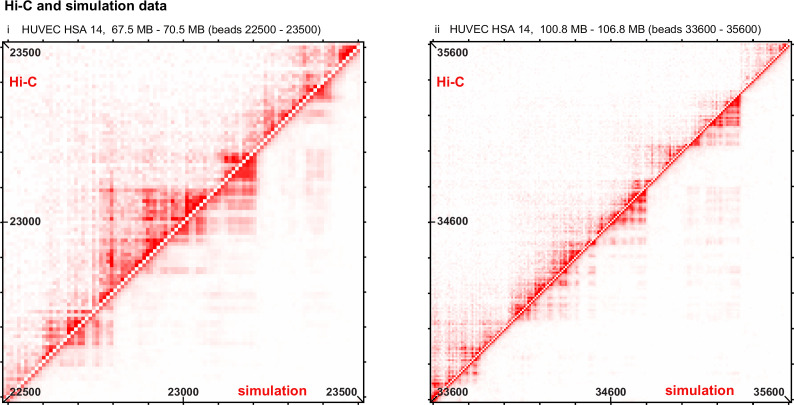
Comparing experimental Hi-C and numerical contact maps for HUVEC HSA 14. After running 100 simulations for HUVEC HSA 14, contact maps for two chromosome fragments are generated and compared to an experimental Hi-C map provided by The ENCODE Project Consortium, 2012. (**i**) fragment 67.5−70.5 MB (beads 22,500−23,500). (ii) fragment 100.8−106.8 MB (beads 33,600−35,500). In both cases the binning is 30 kbp, each pixel is coloured according to the number of contacts and fair data agreement is found (Pearson coefficient $r\sim 0.7$, p-value $ < 10^{-6}$ two-sided t-test). The diagonal is made white for graphical clarity.

Activity predictions are also improved compared to the one-colour model with HSA 18 and HSA 19 in HUVECs, plus HSA 14 in GM12878 (Figure 6D, Figure 6—figure supplement 1). However, Spearman’s rank coefficient for gene-poor HSA 18 is about twice that for gene-rich HSA 19; this may be due to additional regulatory layers in regions with high promoter density. These results show that our multicolour polymer model generates strings that can mimic structures and functions found in whole chromosomes. Additionally, simulated contact maps show a fair agreement with Hi-C data (Figure 6, Figure 6—figure supplement 2), with a Pearson correlation $r\sim 0.7$ (*p*-value <10^−6^, 2-sided t-test). However, because this 2-colour model does not include heterochromatin-binding proteins and cohesin-mediated active loop-extrusion, as in the Hip-Hop model later discussed, we should not expect a very accurate reproduction of Hi-C maps.

### Specialised and mixed clusters

**Figure 7.**
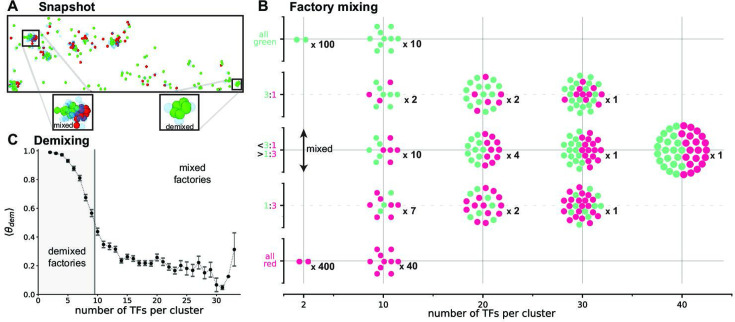
Small clusters tend to be unmixed, large ones mixed. After running one simulation for HSA 14 in HUVECs, clusters are identified. (**A**) Snapshot of a typical final conformation (transcription units, TUs, non-binding beads, and transcription factors (TFs) in off state not shown). Insets: a large mixed cluster and a small demixed one. (**B**) Example clusters with different numbers of TFs/cluster (2, 10, 20, 30, 40) chosen to represent the range seen from all-red to all-green (with 3three intervening bins). Black numbers: observed number of clusters of that type seen in the simulation. (**C**) Average of the demixing coefficient $\theta_{\rm dem}$ (error bars: SD), showing a crossover between demixed and mixed clusters with increasing cluster size. Values of 1 and 0 are completely demixed and completely mixed, respectively. Grey area: demixed regime where $\theta_{\rm dem}$ is >0.5.

Inspection of simulation snapshots shows 1-colour clusters tend to be smaller than mixed (2-colour) ones (Figure 7A). To quantify this, we count numbers and types of TFs in individual clusters (Figure 7B). Clusters with just two bound TFs never contain both colours; conversely, those with >20 bound TFs never contain just one colour (Figure 7B). We also measure the average value of the demixing coefficient, $\theta_{\rm dem}$ (Materials and methods). If $\theta_{\rm dem}=1$ ($\theta_{\rm dem}=0$), this means that a cluster contains only TFs of one colour (a mixture of TFs) and so is fully demixed (maximally mixed). The result is shown in Figure 7C, and shows the emergence of a crossover between a demixed regime, corresponding to single-colour clusters, and a mixed regime, corresponding to multiple-colour clusters, which is triggered by an increase in cluster size. [Note that we speak of a crossover between regimes, rather than a phase transition, as clusters are finite-size and we do not consider any thermodynamic limit.] The crossover point between fully mixed and demixed (where the average value of $\theta_{\rm dem}$ = 0.5) occurs when there are ∼10 TFs per cluster (Figure 7C): notably, this is similar to the average number of productively-transcribing pols seen experimentally in a transcription factory (Cook and Marenduzzo, 2018). Similar results are obtained for different cell types or chromosomes (see Figure 7, Figure 7—figure supplements 1 and 2 for the case of HSA 18, 19 in HUVEC, and HSA 14 in GM12878), and chromosomes under confinement (Figure 7, Figure 7—figure supplement 3), with realistic chromatin densities. The latter situation suggests that, as far as only the formation of transcription factories and the crossover between mixed and demixed clusters are considered, chromatin density does not play a crucial role. Other phenomena can indeed depend on density, especially with respect to global chromatin structure (e.g. entanglements and rare long-range contacts). Additionally, simulations of HSA 14 in HUVEC cells with different size of TF:pols ($0.5\sigma$ and $0.16\sigma$) also lead to similar results (Figure 7—figure supplement 4). Importantly, the critical number of TFs per cluster separating demixed and mixed cluster is around ∼10 in all these different cases. These results suggest that neither the sequence of TUs and its ratio to TFs (which varies among chromosomes, as for instance HSA 18 and HSA 19 are gene poor and gene rich, respectively), nor the chromatin density affect the nature of the crossover between the regimes of demixed and mixed clusters.

**Figure 7—figure supplement 1.**
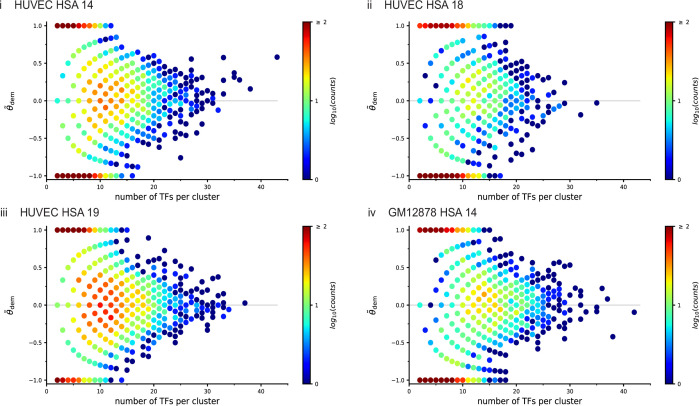
Small clusters tend to be less mixed than large ones in simulations of various human chromosomes in two cell types. After running 100 simulations for each chromosome in each cell type, all clusters are identified, numbers of housekeeping and cell-type-specific transcription units (TUs) in them counted, and demixing coefficients determined. Each dot in a plot is positioned according to TF content and value of $\tilde{\theta}_{\rm dem}=\left(2x_{\rm red}-1\right)$ with $x_{\rm red}$ the fraction of red beads in a cluster (so that $\tilde{\theta}_{\rm dem}=-1$ $\tilde{\theta}_{\rm dem}=-1$ correspond to fully demixed red and green clusters, respectively). Each dot is coloured according to its frequency of occurrence (right-hand bars). (**i**) HSA 14 in HUVEC. (**ii**) HSA 18 in HUVEC. (**iii**) HSA 19 in HUVEC. (**iv**) HSA 14 in GM12878. All panels have similar patterns; each indicates there are high numbers of demixed small clusters (top and bottom left of each panel), and lower numbers of mixed larger clusters (at middle right in each panel).

**Figure 7—figure supplement 2.**
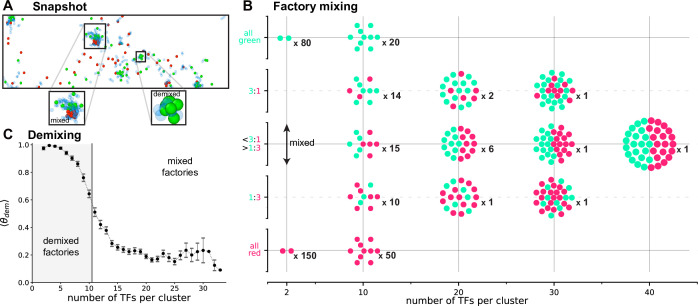
Cluster analysis for HSA 14 in GM12878. After running 100 simulations, clusters are identified. (**A**) Snapshot of a typical final conformation (transcription units, TUs, non-binding beads, and transcription factors TFs, in off state not shown). Insets: a large mixed cluster and a small demixed one. (**B**) Cartoons showing representative contents of clusters. We consider five types of clusters (ranging from all green to all red, $y$ axis), and 5 values for the total numbers of TFs/cluster (2, 10, 20, 30, 40 $x$ axis). Cartoons: cluster composition. Black numbers: observed number of clusters in the simulation. (**C**) Average of the demixing coefficient $\braket{\theta_{\rm dem}}$ as a function of cluster size (error bars: SD). Gray area: demixed regime (corresponding to specialised factories) where $\braket{\theta_{\rm dem}}$ is >0.5. White area: mixed regime (corresponding to mixed factories associated with HOTs) where $\braket{\theta_{\rm dem}}$ is <0.5.

**Figure 7—figure supplement 3.**
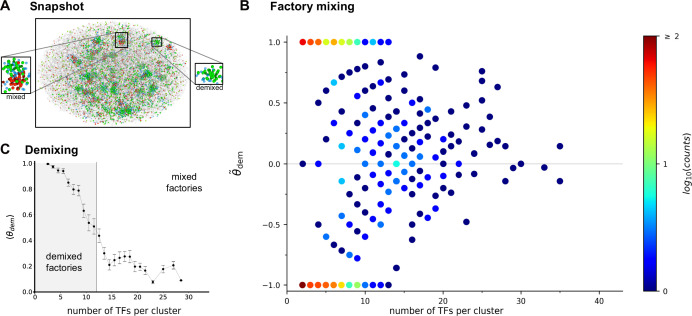
Cluster analysis for confined HSA 14 in HUVEC. After running 100 simulations, clusters are identified for HSA 14 in HUVEC. The system is confined into an ellipsoidal territory with aspect ratio chosen according to typical experimental values (Wang et al., 2017) (semiaxes were $22.24\sigma:34.24\sigma:41.80\sigma$, or $0.67\mu m:1.03\mu m:1.25\mu m$). Confinement is enforced by modifying the source code in LAMMPS to describe an ellipsoidal indenter. This introduces a soft force towards the centre of the ellipsoid, only if beads exit the confining ellipsoid. (**A**) Snapshot of a typical confined conformation. Insets: a large mixed cluster and a small demixed one (non-binding beads and transcription factors, TFs, in off state not shown). (**B**) All clusters are identified, numbers of housekeeping and cell-type-specific transcription units (TUs) in them counted, and demixing coefficients determined. Each dot in the plot is positioned according to TF content and value of $\tilde{\theta}_{dem}$ and coloured according to its frequency of occurrence (right-hand bar). (**C**) Average of the demixing coefficient $\braket{\theta_{dem}}$ as a function of cluster size (error bars: SD). Gray area: demixed regime where $\braket{\theta_{dem}} > 0.5$. White area: mixed regime where $\braket{\theta_{dem}} < 0.5$.

**Figure 7—figure supplement 4.**
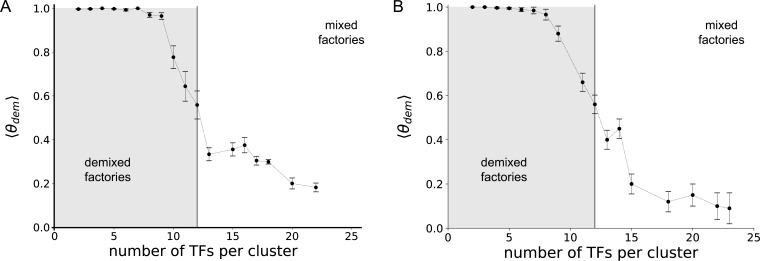
Cluster analysis for HSA 14 in HUVEC with smaller transcription factors (TFs). Clusters are identified for HSA 14 in HUVEC after running 80 simulations with TFs size $0.5\sigma$ (**A**) and 100 simulations with TFs size $0.16\sigma$ (**B**). Panels report average of the demixing coefficient $\braket{\theta_{dem}}$ as a function of cluster size (error bars: SD). Grey area: demixed regime (corresponding to specialised factories) where $\braket{\theta_{dem}} > 0.5$. White area: mixed regime (corresponding to mixed factories associated with HOTs) where $\braket{\theta_{dem}} < 0.5$.

**Figure 7—figure supplement 5.**
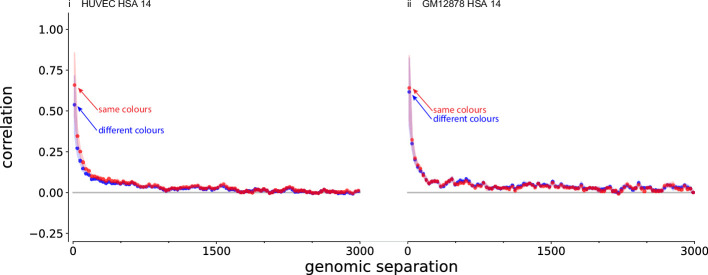
Effect of genomic separation on activity correlations found in whole human chromosomes. Correlation values (shading shows +/- SD, and is usually less than line/spot thickness) at fixed genomic distance are taken from super-/sub-diagonals of Pearson matrices. Red dots give mean correlations between transcription units (TUs) of same colour, and blue dots those between TUs of different colours. (**i**) HSA 14 in HUVEC. (ii) HSA 14 in GM 12878.

**Figure 7—figure supplement 6.**
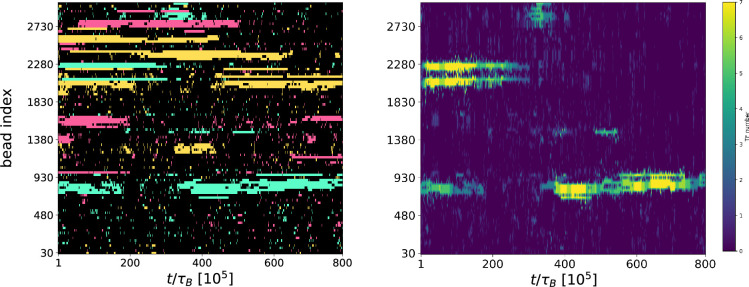
Left: Kymograph showing, at each time step, the transcriptionally active transcription units (TUs), colour-coded according to their assigned type. This kymograph is identical to that reported in Figure 2E (i) of the main text. Right: Corresponding kymograph displaying the number of green transcription factors (TFs) within a sphere of radius $10\,\sigma$ centred on each green TU.

The existence of a crossover between specialised (demixed) and mixed factories with increasing size is, therefore, a generic feature of our model, and it can be explained by the following physical argument depending on non-specific binding. Two red TFs in an unmixed cluster might stabilise three loops, and so bring into close proximity only a few non-specific binding sites that could bind a green TF. In contrast, 10 red TFs in a cluster will stabilise many loops that inevitably bring into close proximity many non-specific binding sites – and this makes it highly likely that some green TFs will also bind nearby to create a mixed cluster. The mixing crossover provides a way to reconcile observations that some clusters are unmixed (like factories rich in polymerases II and III), and others highly mixed (like HOTs). This is because clusters in a single cell are generally polydisperse, or differ in size (e.g. due to the local chromatin environment, or the patterning of TUs along the genome), hence mixed and specialised factories can coexist in the same nucleus. Note that cluster size is a key parameter because it strongly affects the balance between non-specific and specific chromatin-protein interaction.

Finally, as for the toy model, the balance between mixing and demixing determines correlation patterns. For example, activity patterns of same- and differently-coloured TUs in the whole chromosome (Figure 7—figure supplement 5) are much like those in the 1-pattern model (Figure 5Biii). We attribute this to ∼78% TFs being in mixed clusters ($\theta_{\rm dem} < 0.5$), and so inevitably the resulting interactions will dominate the pattern seen.

Our model is already inherently out of thermodynamic equilibrium, as it includes non-equilibrium switching between binding and non-binding states for chromatin-binding proteins, resembling ATP-dependent post-translational modification of such proteins (Brackley et al., 2017b). There are though important principles of chromatin organisation which the presented model does not consider. First, an important remaining question is whether other active (ATP-consuming) processes naturally present in the nucleus, such as loop extrusion (Fudenberg et al., 2016), affect the results we found. Second, following the same logic behind the multicolour polymer model presented here, it is interesting to ask whether the presence of additional types of inactive, as well as active, TFs and chromatin beads changes the picture.

**Figure 8.**
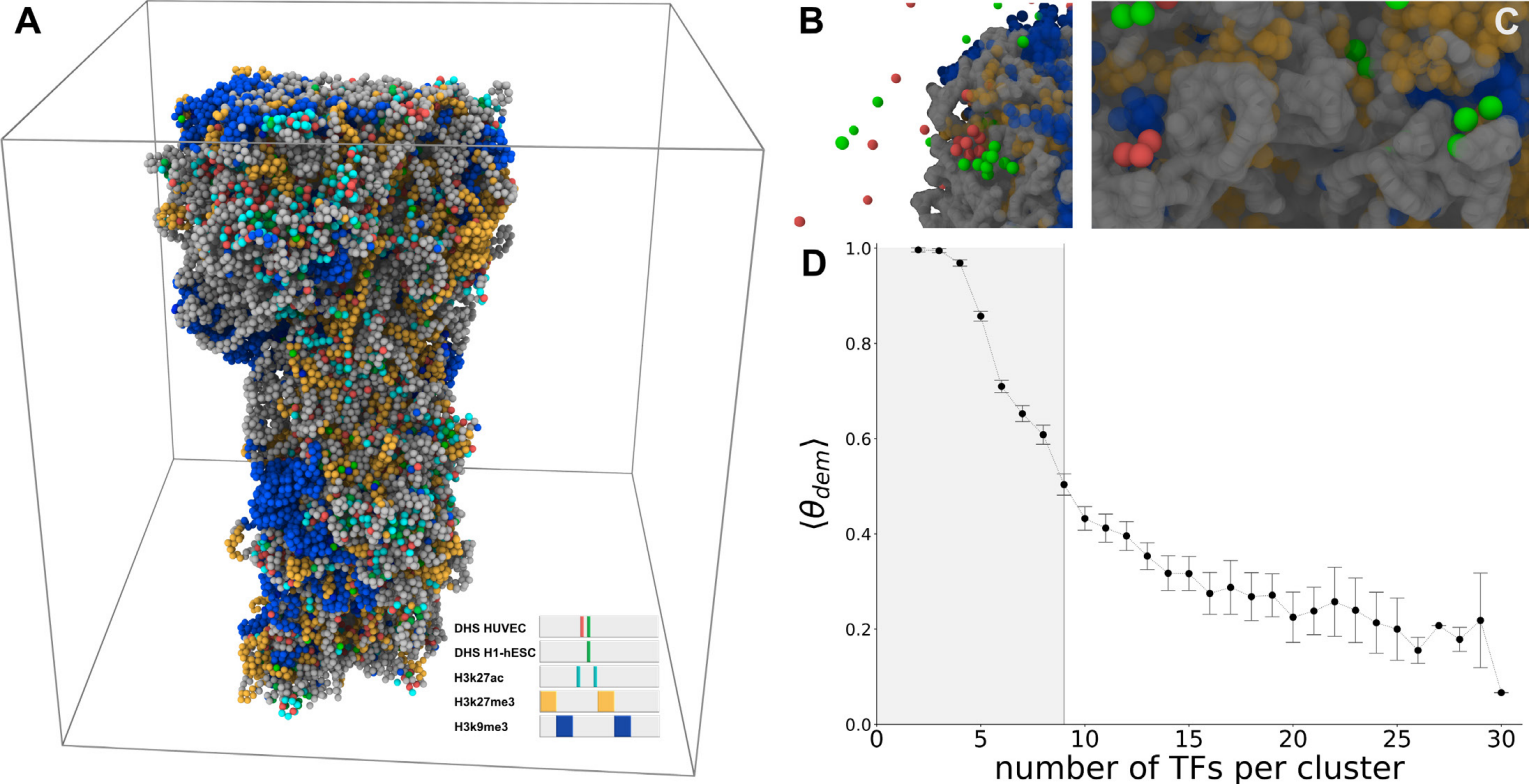
Highly predictive hetero-morphic polymer (HiP-HoP) model simulations: small clusters tend to be unmixed, large ones mixed. (**A**) Snapshot of a configuration adopted by HSA14 in HUVECs, within the HiP-HoP model. Grey regions represent less accessible chromatin regions poor in H3K27ac, while cyan regions represent those enriched in H3K27ac. In addition, H3K27me3 and H3K9me3 peaks determine the chromatin binding sites for polycomb-like and heterochromatin proteins, and are represented in yellow and blue, respectively. As in the previous DNase hypersensitive site (DHS) multicolour model, transcription units (TUs) only present in HUVEC are represented in red, while the house-keeping ones in green. (**B–C**) Example of clusters of proteins: large mixed cluster (**B**) and a small demixed one (**C**). (**D**) Average of the demixing coefficient $\theta_{\rm dem}$ (error bars: SD). Values of 1 and 0 correspond to completely demixed and completely mixed clusters, respectively. Grey area: demixed regime where $\theta_{\rm dem}$ is >0.5.

To answer these questions, we have turned to a more complex framework, and to the HiP-HoP model, which includes loop extrusion by cohesin-like complexes and chromatin heteromorphism (Buckle et al., 2018; Chiang et al., 2022c), as well as accounting for inactive, as well as active, chromatin and TFs. Specifically, we performed simulations for HSA14 in HUVEC using a multicolour version of the HiP-HoP model (see SI for more details). A typical configuration is shown in Figure 8A, where grey regions represent locally compact regions (which are poor in H3K27ac), while cyan regions represent disrupted regions (which are enriched in decompacted chromatin and in H3K27ac). In addition, H3K27me3 and H3K9me3 data were used to determine the chromatin binding sites for polycomb-like and heterochromatin-associated proteins (such as HP1): these are represented in yellow and blue, respectively. As in the previous DHS model, TUs only present in HUVEC are represented in red, while the housekeeping ones in green. Inspection of simulation snapshots shows the presence of small clusters that are demixed (Figure 8C) and large clusters that are mixed (Figure 8B). We also measure the average value of the demixing coefficient, $\theta_{\rm dem}$ (Figure 8D). As in the simpler DHS model, the crossover point between fully mixed and demixed (where the average value of $\theta_{\rm dem}$ = 0.5) occurs when there are ∼10 TFs per cluster. These simulations further confirm the robustness and generality of our results regarding the mixing-demixing crossover between specialised and mixed transcription factories.

## Discussion

In summary, in this paper, we have used coarse-grained simulations to study the 3D structure of human chromatin, its transcriptional dynamics, and their mutual relationship. Unlike previous works (Brackley et al., 2021; Brackley et al., 2016), here we adopt a multicolour model, viz a polymer model in which chromatin interacts with different types (colours) of complexes between polymerases and chromatin-binding transcription factors (TF:pols). This accounts for the important biological fact that most eukaryotic cells show different kinds of RNA polymerases and a variety of chromatin-binding proteins, with different biological scopes. Our model yields a number of experimentally relevant results.

First, we characterise the morphology of transcription factories (or clusters), arising in our model through the bridging-induced attraction (Brackley et al., 2013). When these clusters are small, they typically contain TFs of just one colour; these are reminiscent of the specialised transcription factories found in the nucleoplasm that contain active forms of just pol II or pol III – but not both (Xu and Cook, 2008). Instead, when factories are large, they are typically mixed (Figure 7C); this provides a mechanistic basis for the formation of HOTs, where many different TFs bind promiscuously and weakly to segments of open chromatin that are often devoid of high-affinity specific binding sites (Moorman et al., 2006; Foley and Sidow, 2013; Cortini and Filion, 2018). The existence of a transition (more precisely, a crossover) between demixed and mixed clusters dependent on cluster size is robust to changes in TF:pol size (Figure 7—figure supplement 4), chromatin density (Figure 7—figure supplement 3) and the inclusion of active processes such as loop extrusion, incorporated through the HiP-HoP model (Figure 8). The latter simulations also show that the existence of a demixing transition (and the critical size threshold) is not affected by other structurally important ingredients in the model such as the presence of silence or inactive chromatin, and chromatin heteromorphism (Buckle et al., 2018). This confirms that the mechanism behind the transition is the shift in balance between non-specific and specific chromatin-protein interactions: the former becomes more dominant as cluster size increases. Interestingly, the mechanisms that determine whether a gene belongs to a specialised or mixed factory remain unclear (Razin et al., 2011). However, our results suggest that the TF cluster size, along with the 1D TU patterning along the chromatin filament, plays a crucial role, as it links 3D chromatin structure, transcription factory morphology, and gene expression. Specialised and mixed factories thus emerge naturally from TUs arrangement, without the need for additional ingredients, such as post-transcriptional biochemical regulation.

We propose that the predicted demixing–mixing crossover may be experimentally testable in the future through techniques capable of detecting multi-way chromatin interactions, such as SPRITE and GAM (Kempfer and Pombo, 2020). At present, however, these methods have primarily enabled the detection of three-way chromatin contacts (Quinodoz et al., 2018; Quinodoz et al., 2021; Beagrie et al., 2017; Beagrie et al., 2023), and statistical data on higher-order interactions remain limited or unavailable (Liu et al., 2021). Nevertheless, it is reasonable to expect that ongoing advancements in these technologies will yield increasingly comprehensive datasets, potentially allowing for direct experimental validation of our predictions.

Second, we see remarkable positive and negative correlations in the transcriptional activities of different TUs. For example, activities of same-colour and nearby TUs tend to be strongly positively correlated, as such TUs tend to co-cluster (Figure 5). Conversely, activities of similar TUs lying far from each other on the genetic map are often weakly negatively correlated, as the formation of one cluster sequesters some TFs to reduce the number available to bind elsewhere (Figure 7—figure supplement 6).

Taken together, these results provide simple explanations of why adjacent TUs throughout large domains tend to be co-transcribed so frequently (Hurst et al., 2004), as they are likely to gather together in the same cluster. Results also show how one eQTL might up-regulate some TUs and down-regulate others, that can lead to genetic effects like ‘transgressive segregation’ (Brem and Kruglyak, 2005).

Third, we can predict effects of local mutations and genome edits that often induce distant omnigenic effects uncovered by genome-wide association studies (Boyle et al., 2017; Brackley et al., 2021). For example, mutations that switch a binding site of one TF to another can convert a cluster of one colour into another (Figure 2). Similarly, global effects of knocking down TF levels are easily assessed (Figure 3).

Fourth, we also predict transcriptional activities of all TUs (both genic and non-genic) on whole human chromosomes by including cell-type-invariant and cell-type-specific TFs (Figure 6). We find this yields a better correlation with GRO-seq experimental data than a single-colour model (where just one TF binds to all TUs similarly). This result underscores the importance of including different TFs in polymer models.

Finally, all our results point to the importance of the 1D pattern of TUs and TF-binding sites on chromosomes in determining activity. In other words, 1D location is a key feature determining transcriptional patterns, and so cell identity. We speculate this is why relative locations of active regulatory elements are so highly conserved. For instance, despite human enhancers evolving much more rapidly than their target protein-coding genes, the synteny between the two (over distances up to 2 Mbp) is highly conserved (Berthelot et al., 2018; Laverré et al., 2022).

In the future, it would be interesting to incorporate the effects of transcription elongation into our model. Although the current implementations of both the simplified toy model and the more detailed HiP-HoP model show good agreement with GRO-seq data, further investigation is needed to assess how molecular motors —such as RNA polymerases—and interactions between RNA molecules and nuclear proteins, including SAF-A (Nozawa et al., 2017; Marenda et al., 2024), contribute to the three-dimensional organisation of chromatin. Additionally, it would be valuable to consider the effects of hydrodynamic interactions (Eshghi et al., 2023a; Eshghi et al., 2023b; Mahajan et al., 2022). In our model, as in most chromatin polymer models in the literature (Chiang et al., 2022c), hydrodynamic interactions and the resulting spatiotemporal correlations are neglected. Whilst this choice provides significant computational advantages, it also represents a limitation. Although passive hydrodynamic flow associated with polymer motion is likely screened inside the nucleus, the dipolar forces exerted by molecular motors may be strong enough to induce ordering in the intranuclear polymer melt (Zidovska et al., 2013). In fact, recent experiments using Displacement Correlation Spectroscopy, used to map chromatin movements throughout the nucleus in live cells, revealed that chromatin exhibits rapid, uncorrelated motions at short timescales and slower, correlated motions over ∼*μ*m domains at longer timescales (Zidovska et al., 2013). While the typical sizes of the emerging clusters we observe are about an order of magnitude smaller than that of these domains, incorporating hydrodynamics would help elucidate the effect of coherent chromatin dynamics on cluster formation and coarsening.

In addition, it would be of interest to extend the results presented here to incorporate many more types of TFs and TUs within the framework of HiP-HoP (Buckle et al., 2018), and to include dynamic epigenetic modifications (Brackley et al., 2017b; Michieletto et al., 2016; Olarte-Plata et al., 2016). From a theoretical point of view, we hope our results will stimulate the development of theories to understand the mixing-demixing crossover more fundamentally from a polymer physics viewpoint, as well as more work on the interrelations between 3D structure and function in chromosomes.

## Methods

Chromatin fibres are modelled as bead-and-spring polymers (Brackley et al., 2013; Brackey et al., 2020; Brackley et al., 2021; Buckle et al., 2018; Bianco et al., 2017; Brackley et al., 2017a; Brackley et al., 2016; Nicodemi and Pombo, 2014; Conte et al., 2022; Semeraro et al., 2023b; Tiana et al., 2016; Giorgetti et al., 2014; Crippa et al., 2020; Semeraro et al., 2023b; Natesan et al., 2022), where each monomer (diameter σ) represents 3 kbp packed into a 30 nm sphere Brackley et al., 2021; Brackley et al., 2013; Semeraro et al., 2023b. Different TFs (or TF:pol complexes) are modelled as differently-coloured spheres (also with diameter σ) able to bind (when in an ‘on’ state) to cognate sites of the same colour that are scattered along the polymer. TF:pols complexes and TUs have the same size, since the former represents both transcription factors and polymerases, which in human cells are about 5 nm and 25 nm, respectively. We also simulated the case in which TF:pol size is smaller ($0.5\sigma$ and $0.16\sigma$, Fig. S9) to explore the potential effect of protein size.

Each TF and TF:pol switches between ‘off’ (non-binding) and ‘on’ (binding) states to reflect the post-translational modifications that occur in many TFs. Polymer beads are either non-binding (‘heterochromatic’), weakly-binding (‘euchromatic’), or strongly-binding (containing cognate sites). TFs bind non-specifically to all weakly-binding beads, and strongly only to TUs of the same color. TUs in our model represent regulatory elements, such as promoters and enhancers, and as discussed below in practice can be identified with DNase hypersensitive regions, which are very sticky for a wide range of TFs, or active protein complexes (The ENCODE Project Consortium, 2012).

The system evolves in a cubic simulation domain with periodic boundary conditions through constant-temperature Langevin dynamics that are integrated numerically by the LAMMPS simulation package (Thompson et al., 2022).

In our model, as in most chromatin polymer models in the literature (Chiang et al., 2022a), hydrodynamic interactions are neglected. While this choice offers significant computational advantages, it also presents a limitation. Although passive hydrodynamic flow associated with polymer motion is likely screened inside the nucleus, dipolar forces exerted by molecular motors may still be strong enough to induce ordering in the intranuclear polymer melt, as discussed in Zidovska et al., 2013 (see also the Discussion section for additional comments on the possible effects of hydrodynamics). Averages are evaluated over 100 independent runs for each case. The TF volume fraction of each colour is set to ∼3×10^−5^, and the polymer volume fraction to ∼2×10^−3^. We note though that the key control parameter is the ratio between the number of TFs and that of TUs, for each colour. More information about the model can be found in Appendix 1.

Several quantities are monitored to describe the system’s behaviour. Mean transcriptional activity is measured as the fraction of time that a TU is ‘transcriptionally active,’ i.e., within a fixed distance of a TF, in 100 simulations, and so represents a population average (each simulation run may be thought of as a different cell). In order to be quite conservative about the evaluation of the transcriptional activity for TUs in clusters, we adopt $2.25\sigma$ as transcriptional threshold. Our point of view is in fact to consider transcriptionally active all TUs in clusters, even when TFs are a bit further. However, we also checked that lower distance thresholds do not affect evaluation of transcriptional profiles in any significant way (see Figure 1—figure supplement 1 in SI). Transcriptional activity is then compared with experimental data on transcriptional activity, obtained via GRO-seq – a method providing a genome-wide average readout of ongoing transcription of both genic and non-genic TUs in cell populations (Core et al., 2014; Jordán-Pla et al., 2019). The mean transcriptional Pearson correlation between all pairs of TUs is also evaluated, and a graphical overview of this feature is provided via the Pearson correlation matrix. We also analyse clusters/factories of bound and spatially-proximate TFs, count the number of TFs of similar colour in each cluster, and introduce a demixing coefficient

(1) $$\displaystyle   \theta_{\rm dem}=\frac{nx_{i,max}-1}{n-1}, $$

where $n$ is the number of colours, $i$ is an index denoting each of the colours present in the model and $x_{i,max}$ the largest fraction of TFs of the same $i$-th colour in a single TF cluster. If $\theta_{\rm dem}=1$, this means that a cluster contains only TFs of one colour and so is fully demixed; if $\theta_{\rm dem}=0$, the cluster contains a mixture of TFs of all colours in equal number, and so is maximally demixed. More details can be found in the SI.

We consider two different types of string, one with $M=3000$ beads (or 9 Mbp) which is referred to as a ‘toy’ string, and a second representing a whole human chromosome. Chromosomes are initialized in both cases as random walks. An alternative possibility would be to start from mitotic configuration as in Rosa and Everaers, 2008, which would remove entanglement in the initial condition. Experience with similar models (e.g. see Jost et al., 2014) suggests that a different initial condition will be important for the very large-scale structure but not for the scale at which transcriptional clusters form, which is the one we are most interested in here.

### Toy model

The toy model is built by placing one yellow, red, or green TU every 30 weakly-binding beads, giving a total of 100 TUs of all types in a string of 3000 beads (Brackley et al., 2013). Various different sequences of TU colour down the string are considered. In one – the ‘random’ string – TU colours are chosen randomly (see Figure 1a and SI for the specific sequence generated). In a second and third – the ‘1-pattern’ and ‘6-pattern’ strings – TU colours follow a repeating pattern (red, then yellow, then green) 1 or 6 times (see Figure 4). We made these choices for the sequences of TUs as they are useful to show how 1D patterns affect resulting cluster morphology. In this respect, these patterns in the toy model are only representative. At the same time, the ratio between TFs and TUs are close to those used below for human chromosome simulations.

For the random string, we monitor how the system responds to different perturbations. Local ‘mutations’ are inspired by editing experiments performed using CRISPR/Cas9 Morgan et al., 2017. One to four mutations are mimicked by switching selected yellow beads inside a cluster of consecutive yellow TUs (between TUs 1920 to 2070) to red ones (Figure 2). Thus, conversion of TU bead 1980 gives a string with one mutation, of 1950 and 1980 gives two mutations, of 1950 to 2010 gives three mutations, and 1950 to 2040 gives four mutations. Global perturbations are inspired by experiments reducing global levels of TFs using auxin-induced degrons (Luan et al., 2021). Here, we study the effects of reducing the concentration of yellow TFs by 30%.

### Human chromosomes

Our reference case for whole human chromosome simulations in the main text is the mid-sized human chromosome HSA 14 (107 Mbp), coarse-grained into $M=35784$ beads. For Figure 6, weakly- and strongly-binding beads are identified (using The ENCODE Project Consortium, 2012 for human umbilical vein endothelial cells, HUVECs) by the presence of H3K27ac modifications and DNase-hypersensitivity sites (DHSs) in the 3 kbp region corresponding to that bead – as these are good markers of open chromatin and active TUs (both genic and non-genic), respectively (see Appendix 2 for more details about the use of epigenetic marks). For Figure 6, TUs are split into ones only active in HUVECs and others (‘house-keeping’ ones) that are also active in H1-hESC cells (again using DHS sites and ENCODE data). Then, if a TU appears in both HUVECs and H1-hESCs, it is marked as housekeeping and coloured red; if it appears only in HUVECs it is marked as HUVEC-specific and coloured green. This allows an intuitive and simple multicolour model of HUVECs to be constructed. All remaining beads (which are not either weakly-binding or TUs) are non-binding. This approach represents a generalisation of the DHS model described in Brackley et al., 2021, so we call it the multicolour DHS model. For the simulations shown in the main text TF:pols complexes and TU size is the same (σ, corresponding to 30 nm at our resolution). This is justified by the fact that our TF:pol represents both transcription factors and polymerases. A polymerase is about 25 nm in human cells (Cook, 2001), while transcription factors are typically at least 5 nm in size. We also considered the case in which TF:pol size is smaller ($0.5\sigma$ and $0.16\sigma$, Figure 7—figure supplement 4) to explore the potential effect of protein size: as we shall see, this does not qualitatively affect our conclusions and results.

We also consider HSA 18 (80 Mbp, 26026 beads) and 19 (58 Mbp, 19710 beads) in HUVECs, chosen as they represent gene-poor and gene-rich chromosomes, respectively. Additionally, we consider HSA 14 in the B-lymphocyte line GM12878 (again, colours are chosen by combining DHS data for GM12878 and H1-hESCs). H3K27ac and DHS data is again from ENCODE.

The multicolour DHS model was also applied within a more realistic chromatin framework, the ‘highly predictive heteromorphic polymer model,’ or HiP-HoP model (Buckle et al., 2018). This is a much more sophisticated model which takes into account: (i) loop extrusion; (ii) inactive (as well as active) chromatin folding; (iii) chromatin heteromorphicity (different local compaction of chromatin according to acetylation). More details on the HiP-HoP model are given in Appendix 3.

For human chromosomes, transcriptional-activity data obtained from simulations and GRO-seq are compared in two ways (Brackley et al., 2021). First, we rank activities of each TU and build a two-dimensional histogram providing an overview of the agreement between the two sets of ranks. Second, we quantify Spearman’s rank correlation coefficient between numerical and experimental data.

## Data Availability

All experimental data used in this paper are available in the ENCODE database (Hiram, 2018). All custom scripts used for the simulations presented here are available at Zenodo (https://doi.org/10.5281/zenodo.17408464; Negro, 2026).

## Additional files

MDAR checklist

## Appendix 1

### Simulation details

#### Multicolour bead polymer model

Chromatin filaments are coarse-grained into bead-and-spring polymers with $M$ monomers (Brackey et al., 2020), each monomer having a diameter of $\sigma=30$ nm, corresponding to a 3 kbp genomic region (Brackley et al., 2013) (a slightly different mapping from genomic to spatial distances would not affect the final results of our work Florescu et al., 2016). There are three types of beads: $M_{nb}$ beads interacting repulsively with all TFs and so non-binding, $M_{w}$ beads weakly binding TFs, and $\{M_{i}\}\, =1,\ldots,n_{c}$ beads strongly binding to same-coloured TFs only; $n_{c}$ here denotes the number of TU colours. Thus, the total number of chain beads is $M=M_{nb}+M_{w}+\sum_{i=1}^{n_{c}}M_{i}$. TFs are also modelled as spheres with the same diameter σ; they can either be in a binding (on) or non-binding (off) state. In the on state, TFs can bind strongly to TUs of the same colour, while in the off state they do not bind. At every time interval, $t_{s}$, each TF can change its state becoming off from on, or vice versa, with rates $\alpha_{off}$ and $\alpha_{on}$, respectively. The total number of TFs is $N=\sum_{i=1}^{n_{c}}2N_{i}$, where the number of TU and TF colours is the same and the factor of 2 takes into account the on/off counting (for each colour, $N_{i}$ TFs are initially set on, and $N_{i}$ initially off). Finally the total number of beads is $M+N$, the position of each monomer (either TFs or chain monomers) is denoted as $\textbf{r}_{i}=(r_{ix},r_{iy},r_{iz}),\, i=1,\ldots,M+N$, and the distance between the generic *i*-th and *j*-th beads is denoted as $r_{ij}$.

To ensure chain connectivity, two consecutive chain beads $i$ and $j=i+1$ interact through the harmonic potential:

$$\displaystyle   U_{harmonic}=-k_{h}\left(r_{ij}-\bar{r}\right)^{2}~,$$

where $k_{h}=100k_{B}T/\sigma^{2}$ is the harmonic spring constant, $\bar{r}$ is the equilibrium spring distance set at $\bar{r}=1.1 \sigma$, $k_{B}$ is the Boltzmann constant, and $T$ is temperature. Chain stiffness is described via a bending (Kratky-Porod) potential depending on the position of every three consecutive chain beads as follows:

$$\displaystyle   U_{KP}=\frac{k_{B} T l_{P}}{\sigma}\left[1-\frac{\vec{s_i}\cdot\vec{s_j}}{|\vec{s_i}||\vec{s_j}|}\right]=k_{kp}\left[1-\cos(\theta)\right]~,$$

where $i$ and $j=i+1$ are neighbouring polymer beads, $\vec{s_{i}}$ is the tangent vector connecting the $i$-th and the $i+1$-th beads, $\theta$ is the angle formed by such tangents, and $l_{P}$ is the persistent length of the chain.

Any two beads $i$ and $j$ interact in general through a truncated and shifted Lennard-Jones potential:

$$\displaystyle   U_{LJ}= \begin{cases}4\epsilon_{ij}\left[\left(\frac{\sigma}{r_{ij}}\right)^{12}-\left(\frac{\sigma}{r_{ij}}\right)^{6}-c\right]&\mathrm{~if~}r_{ij} < r_{c}\\ 0&\mathrm{otherwise}\end{cases}~,$$

with $c=\left(\frac{\sigma}{r_{c}}\right)^{12}-\left(\frac{\sigma}{r_{c}}\right)^{6}$, and $r_{c}$ is the interaction cut-off. Different cut-offs and interaction strengths are set depending on the chain bead type and colour, and TF colour, of the two interacting beads. We use $r_{c}=1.8\sigma$ and $\epsilon_{ij}=8k_{B}T$ for strong binding between TUs and on TFs of the same colour, $r_{c}=1.8\sigma$ and $\epsilon_{ij}=3k_{B}T$ for weak binding between chain beads and all on TFs, and $r_{c}=2^{1/6}\sigma$ (purely repulsive potential) and $\epsilon_{ij}=k_{B}T$ for non-binding interactions between non-binding polymer beads and TFs, between pairs of TFs, between pairs of TUs, and between pairs of different-colour TU and TF beads.

A TU bead is considered transcriptionally active when it is bound to at least one same-coloured TF, i.e., when their distance is smaller than a fixed threshold. In order to be quite conservative about the evaluation of the transcriptional activity, here we consider $2.25\sigma$ as such a threshold. However, we checked that varying it within reasonable limits (we tested $1.8\sigma$ and $1.1\sigma$, see Figure 1—figure supplement 1) does not affect the output transcriptional profiles in any significant way.

The time evolution of the entire system is described by the following system of $3(M+N)$ Langevin equations

(2) $$\displaystyle   m_{l}\frac{d^{2} r_{l\beta}}{dt^{2}}=-\nabla U_{l}-\gamma_{l}\frac{d r_{l\beta}}{dt}+\sqrt{2k_{B}T\gamma_{l}}~\eta_{l\beta}(t)~, $$

where $l$ is the bead index running from 1 to $M+N$, $\beta=x,y,z$ is the dimension index, $m_{l}$ and $\gamma_{l}$ respectively are the mass and friction coefficient associated to the $l$-th bead, $U_{l}$ is the total potential experienced by the $l$-th bead, and $\eta_{l\beta}$ are a set of zero-mean delta-correlated stochastic white noises, in symbols

$$\displaystyle \left\lt\eta_{l\beta}(t)\right>=0, \qquad \left\lt{\eta_{l\beta}(t)\eta_{l^{\prime}\beta^{\prime}}(t^{\prime})}\right\gt=\delta_{ll^{\prime}}\delta_{ \beta\beta^{\prime}}\delta(t-t^{\prime}) ,$$

where $\delta_{ll^{\prime}}$ and $\delta_{\beta\beta^{\prime}}$ are two Kronecker deltas and $\delta(t-t^{\prime})$ is a Dirac delta. For the sake of simplicity, we set the mass and friction coefficient equal for all beads, $m_{l}\equiv m$ and $\gamma_{l}\equiv\gamma$, respectively.

### Simulation units and system constants

As typically done in the literature (Brackley et al., 2021), we introduce the Lennard-Jones time $\tau_{LJ}=\sigma\sqrt{m/\epsilon}$, with $\epsilon=k_{B}T$ the energy unit, and the Brownian time $\tau_{B}=\sigma^{2}/D$, with $D=k_{B}T/\gamma$ the diffusion coefficient of a single bead, representing the characteristic time a bead takes to diffuse across its own diameter. Our simulations are performed by setting mass $m$, diameter σ, temperature $T$, and Boltzmann constant $k_{B}$ to one. We also set the friction coefficient $\gamma=1$, so that $\tau_{LJ}=\tau_{B}$, and $k_{kp}/k_{B}T=l_{P}=3\sigma$, making the fibre relatively flexible. The simulation reduced units are then σ for the length, $\epsilon$ for the energy, and $\tau_{B}$ for the time.

Protein switching is implemented by stochastically changing the TF state of all colours every $t_{s}=10^{2}\tau_{B}$ with probability such that the switching off rate is $\alpha_{off}=10^{-5}\, \tau_{B}^{-1}$. Both in the toy model and chromosome simulations the switching on rate is instead $\alpha_{on}=\alpha_{off}/4$, so that in the steady state for each colour the average number of off TFs is 4 times the number of the on ones.

The system is free to evolve in a cubic simulation box with periodic boundary conditions of side $L=173\sigma$ for the toy model, $L=570\sigma$ for HUVEC and GM12878 HSA14, $L=500\sigma$ for HUVEC HSA18 and $L=556\sigma$ for HUVEC HSA19 so that in all cases the system is in the dilute regime and the TU density is the same in all cases. In all toy model cases the initial number of TFs per colour is set at 25, except for the edited one where the initial number of yellow on and off TFs is reduced by 30%, from 25 to 17.

### Mapping to physical units

Conversion from simulation to physical units is readily obtained. Using the realistic values of bead diameter σ = 3×10^–8^ *m* (representing also chain thickness), room temperature $T=300\leavevmode\nobreak\ K$ (giving $\epsilon\simeq 4.14\,\times 10^{-21}\, J$) and Stokes friction coefficient for spherical beads $\gamma=3\pi\eta_{sol}\sigma$, with nucleoplasm viscosity $\eta_{sol}=10-100\, cP$, one finds that $\tau_{LJ}=\tau_{B}=\sigma^{2}/D=3\pi\eta_{sol}\sigma^{3}/\epsilon\simeq 0.6-6 \,\times 10^{-3}\, s$.

### Simulation details

We use the LAMMPS (Large-scale Atomic/Molecular Massively Parallel Simulator) software package Thompson et al., 2022 to simulate the system. For computational efficiency, hydrodynamic interactions are neglected. The system of equations Equation 2 is integrated using the velocity Verlet algorithm. The timestep is set to $\Delta t=0.01\tau_{B}$.

To begin simulations, the chromatin fibre is initialised as a random walk while TFs are placed randomly within the simulation box. Overlapping beads in the initial conformation are relaxed evolving the system for a few timesteps with a soft potential between beads. The system is then thermalised for $10^{5}\tau_{B}\simeq 60-600 s$ for the toy model and for $4\,\times 10^{4}\, \tau_{B}\simeq 24-240 \, s$ for whole chromosomes, considering only repulsive interaction between all couples of beads. Finally, the system is evolved, including all attractive interactions for $8\, \times 10^{5}\, \tau_{B}\simeq 480-4800 \, s$ for the toy model and for $2 \times 10^{5} \tau_{B}\simeq 120-1200\, s$ for chromosomes. For each considered case, we run at least 100 independent simulations.

### Toy model TU colour sequences

We report here the TU colour sequences (bead indexes) of all cases considered for the toy model.

Reference case.
Red TU beads: 60, 210, 300, 360, 390, 480, 630, 660, 720, 990, 1080, 1110, 1170, 1200, 1350, 1380, 1410, 1440, 1530, 1560, 1590, 1620, 1650, 1800, 1890, 2190, 2310, 2460, 2520, 2670, 2730, 2760, 2790, 2910;
Yellow TU beads: 30, 120, 570, 930, 1050, 1140, 1230, 1260, 1290, 1320, 1710, 1770, 1860, 1920, 1950, 1980, 2010, 2040, 2070, 2130, 2160, 2220, 2340, 2370, 2400, 2430, 2550, 2580, 2610, 2700, 2970;
Green TU beads: 90, 150, 180, 240, 270, 330, 420, 450, 510, 540, 600, 690, 750, 780, 810, 840, 870, 900, 960, 1020, 1470, 1500, 1680, 1740, 1830, 2100, 2250, 2280, 2490, 2640, 2820, 2850, 2880, 2940, 3000;

$n$-mutant cases.
Same TU colour sequence as reference case except for yellow→red mutations of the following TU beads: 2010 for 1-mutant case; 1980 and 2010 for 2-mutants case; 1950, 1980 and 2010 for 3-mutants case: 1950, 1980, 2010 and 2040 for 4-mutants case;

6-pattern case.
Red TU beads: 30, 60, 90, 120, 150, 180, 570, 600, 630, 660, 690,720, 1110, 1140, 1170, 1200, 1230, 1260, 1650, 1680, 1710, 1740, 1770, 1800, 2190, 2220, 2250, 2280, 2310, 2340, 2730, 2760, 2790, 2820, 2850, 2880;
Yellow TU beads: 210, 240, 270, 300, 330, 360, 750, 780, 810, 840, 870, 90, 1290, 1320, 1350, 1380, 1410, 1440, 1830, 1860, 1890, 1920, 1950, 1980, 2370, 2400, 2430, 2460, 2490, 2520, 2910, 2940, 2970, 3000;
Green TU beads: 390, 420, 450, 480, 510, 540, 930, 960, 990, 1020, 1050, 1080, 1470, 1500, 1530, 1560, 1590, 1620, 2010, 2040, 2070, 2100, 2130, 2160, 2550, 2580, 2610, 2640, 2670, 2700.

1-pattern case.
Red TU beads: 30, 120, 210, 300, 390, 480, 570, 660, 750, 840, 930, 1020, 1110, 1200, 1290, 1380, 1470, 1560, 1650, 1740, 1830, 1920, 2010, 2100, 2190, 2280, 2370, 2460, 2550, 2640, 2730, 2820, 2910, 3000;
Yellow TU beads: 60, 150, 240, 330, 420, 510, 600, 690, 780, 870, 960, 1050, 1140, 1230, 1320, 1410, 1500, 1590, 1680, 1770, 1860, 1950, 2040, 2130, 2220, 2310, 2400, 2490, 2580, 2670, 2760, 2850, 2940;
Green TU beads: 90, 180, 270, 360, 450, 540, 630, 720, 810, 900, 990, 1080, 1170, 1260, 1350, 1440, 1530, 1620, 1710, 1800, 1890, 1980, 2070, 2160, 2250, 2340, 2430, 2520, 2610, 2700, 2790, 2880, 2970;

single-colour case.
TU beads: 30, 60, 90, 120, 150, 180, 210, 240, 270, 300, 330, 360, 390, 420, 450, 480, 510, 540, 570, 600, 630, 660, 690, 720, 750, 780, 810, 840, 870, 900, 930, 960, 990, 1020, 1050, 1080, 1110, 1140, 1170, 1200, 1230, 1260, 1290, 1320, 1350, 1380, 1410, 1440, 1470, 1500, 1530, 1560, 1590, 1620, 1650, 1680, 1710, 1740, 1770, 1800, 1830, 1860, 1890, 1920, 1950, 1980, 2010, 2040, 2070, 2100, 2130, 2160, 2190, 2220, 2250, 2280, 2310, 2340, 2370, 2400, 2430, 2460, 2490, 2520, 2550, 2580, 2610, 2640, 2670, 2700, 2730, 2760, 2790, 2820, 2850, 2880, 2910, 2940, 2970, 3000;

## Appendix 2

### Inserting epigenetic data

We detail here the procedure used to build the multicolour polymer bead sequences of the chromosomes. The procedure consists of two steps:

1. *Extraction of the bead sequence from experimental data for a single cell line*. ChIP-seq for H3K27ac data and DNase-hypersensitivity (DHS) data for the cell line of interest are used to determine the weakly-binding and TU bead positions along the chain, respectively.
  Concerning H3K27ac, two different unfiltered and control data sets are first downloaded from The ENCODE Project Consortium, 2012 as. bam files and then combined into a main and control. bed files using bedtools (Quinlan and Kindlon, 2023). The tool epic Stovner and Sætrom, 2019 is then employed to identify experimental peaks and produce the final. bed H3K27ac peak file. Peak identification by the epic tool works as follows: first the tool resolves main and control data at fixed resolution (in our case 200 bp); then it compares each resolved region of main and control data and assigns a peak if the amount of ChIP signal in the main data is significantly higher than the amount in the control data using as discriminant criterion the false discovery rate cutoff (in our case 0.05).
  Concerning DHSs, data are first downloaded from ENCODE as .bam files and, as before, two different data sets are taken into account. The peaks from alignment results are then identified and combined through the MACS (Zhang et al., 2008) tool as for the H3K27ac data and cast into the human chromosome size through the bedGraphToBigWig conversion tool project (The ENCODE Project Consortium, 2012), the latter action being performed using as a reference for chromosome size the common hg19.chrom.sizes file hg1 (http://hgdownload.cse.ucsc.edu/goldenpath/hg19/bigZips/hg19.chrom.sizes). The final DHS peak.bed file is then produced by considering only significant peaks (in our case $-\log_{10}(peak\, value)\geq 30$).
  Finally the bead polymer sequence is produced by casting the .bed H3K27ac and DNase-hypersensitivity files into the cell line sequence as prescribed by the chosen polymer bp resolution.
2. *Specific cell line multicolour sequence generation*. The actual multicolour bead polymer sequence for a specific cell line is generated by combining bead sequences for the cell of interest and for the stem cell H1-hESC generated as prescribed by the previous step. Specifically, weakly-binding and TU bead attribution is performed by looking first at the specific cell bead sequences: if a bead contains an H3K27ac peak and no DHS peaks, it is marked as weakly-binding, while DHS peaks are marked as TUs. TU colours are instead determined by combining the specific cell line and H1-hESC TU bead sequences: if a TU appears in both the specific and H1-hESC cell, it is marked as housekeeping, while if a TU appears in the specific cell line sequence only it is marked as specific.

## Appendix 3

### Hip-hop simulations

#### HiP-HoP simulations of HSA 14 in HUVEC

In the main text, we present results regarding the mixing-demixing transition using the *highly predictive heteromorphic polymer model* or HiP-HoP (Buckle et al., 2018) model (main Figure 8). We present here the details, parameters, and input data of this model. In the HiP-HoP model, the chromatin fibre is heteromorphic, meaning that its thickness is not constant. Indeed, in order to catch the less accessible chromatin structure of regions poor in H3K27ac marks (Buckle et al., 2018), the polymer segments without this mark are modelled as crumpled by adding some springs. In particular, if a triplet of beads $i$, $i+1$, and $i+2$ do not include H3K27ac marks, a spring is applied between next-nearest neighbour beads ($i$ and $i+2$) and it is described by the following harmonic potential

$$\displaystyle V_{\rm HARM}=K_{\rm HARM}(r-R_{\rm HARM})^{2},$$

with $K_{\rm HARM}=200\epsilon/\sigma^{2}$ being the spring constant and $R_{\rm HARM}=1.1\sigma$ the equilibrium bond length. In the HiP-HoP model, we include a loop extrusion mechanism which is considered to be performed by the cohesin complex (Fudenberg et al., 2016; Goloborodko et al., 2016; Banigan and Mirny, 2020). The action of cohesin rings is modelled by inserting additional spring bonds between beads; the pair of beads bonded by these springs is then moved outwards, i.e., from $(i,i+3)\rightarrow(i-1,i+4)\rightarrow(i-2,i+5)$ etc., to extrude a loop. Extruders (i.e. cohesin bonds) are loaded on the polymer at random $i,i+3$ positions with rate $k_{\rm on}$ and they unbind with rate $k_{\rm off}$. Their positions are moved at rate $k_{\rm ex}$. The spring potential is given by

$$\displaystyle U_{\rm EXTR}(r_{i,j})=U_{\rm WCA}(r_{i,j})+K_{\rm EXTR}(r_{i,j}-r_{0})^{2},$$

where $K_{\rm EXTR}=40k_{B}T/\sigma^{2}$ is the bond strength and $r_{0}=1.5\sigma$ is the equilibrium spring length. The two ends of each extruder move independently and each of them is halted either when it collides with the end of another extruder, or when it reaches a CTCF site whose direction is opposite to its direction of travel. If an extruder is halted on one end, the position at the other end keeps moving, extruding the loop. The loop extrusion dynamics is obtained by using a Python script using the LAMMPS library.

Different from the previously discussed polymer model, four species of transcription factors (TFs), represented by spheres of the same size as the polymer beads, are inserted in the model, two active, as in the previous model, and two new inactive species. The two active TF types correspond again to general active complexes in charge of transcription of genes (Cook and Marenduzzo, 2018) specific to HUVEC cells and of genes shared by HUVEC and H1-hesc cells, respectively. The two new inactive TF species model polycomb repressive complexes (PRCs) (Boyle et al., 2020) and heterochromatin proteins (such as HP1) (Strom et al., 2017). The interactions between TFs and polymer beads are described by the same shifted and truncated Lennard-Jones potential discussed before. The new inactive TFs experience the following potentials: polycomb-like proteins are highly attracted ($\varepsilon=8\,k_{B}T$) to chromatin beads enriched in H3K27me3, while heterochromatin TFs experience a strong attraction ($\varepsilon=8\,k_{B}T$) to chromatin beads containing H3K9me3 marks.

Finally, to facilitate the phase separation between euchromatin and heterochromatin, an additional weak chromatin-chromatin attraction is inserted between beads not provided with H3K27ac marks. This last potential is described by the shifted and truncated Lennard-Jones potential $V_{LJ/cut}$ with $\varepsilon=0.4\,k_{B}T$. As in the previous model, during the simulations TFs switch back and forth between a binding and a non-binding state with at rate $k_{\rm switch}$ (Brackley et al., 2017b). This represents post-translation modifications which alter the protein-DNA binding affinity (e.g. phosphorylation). When in the non-binding state the interaction between proteins and chromatin binding sites revert to the WCA potential.

We simulate chromosome 14 in HUVEC cells, which, by using a resolution of $3\,\sigma$, is composed of 35,784 beads. The polymer is immersed in a cubic simulation box of side $84.5\,\sigma$ with periodic boundary conditions. This results in a chromatin density of $\sim 60\,bp/\sigma^{3}$, which is the same order of magnitude as in vivo, once mapped to real units (see ‘Mapping simulation units to real units’). The number of active TFs is set equal to 1200 (half in the binding state and half in the non-binding state), both for HUVEC-specific TFs and for TFs interacting with DHS sites found both in HUVEC and H1-hESC cells. We insert then 400 polycomb-like TFs (half in the binding state) and 1000 HP1-like proteins (again, half binding the chromatin fibre).

As initial condition, we generate the polymer as a random walk within the simulation box and we place TFs in random positions. We let the system to relax, initially inserting soft potentials to displace overlapping beads and then by using WCA steric interactions for non-bonded beads. A further equilibration is performed after inserting the additional crumpling springs between proper beads. At this point we create the initial configuration for HiP-HoP simulations by assigning a type to each polymer bead. The bead type depends on the combination of DHS peaks, H3K27ac, H3K27me3, and H3K9me3 marks present in the genome region covered by the bead. HiP-HoP simulations are performed by inserting 1000 extruders, which provide an extruder density of ∼10 extrudes Mbp, comparable to the one observed in vivo.

We run 40 independent HiP-HoP simulations, each for a total time $5\,\times 10^{5}\,\tau_{B}$. We discard the first $10^{5}\tau_{B}$ (by which the system reaches a steady state), so that the analysis is performed on the last $4\times 10^{5}\tau_{B}$.

As in our previous model, in HiP-HoP simulations lengths are given in multiples of the polymer bead diameter σ. As we assume each bead contains $\sim 3\,kbp$, we set $\sigma=30\,\rm nm$. The simulation time unit is defined as the Lennard-Jones time $\tau_{LJ}$. To map the latter, we consider the other two characteristic times of the system: the Brownian time $\tau_{B}$ and the decorrelation time $\tau_{dec}$.

As discussed above and in the main text, TFs switch back and forth between a binding and non-binding state. The other simulation rates are related to loop extrusion and are set as: $k_{on}=2\times 10^{-2}\,\tau_{B}^{-1}$, $k_{off}=5\times 10^{-5}\,\tau_{B}^{-1}$, and $v_{ex}=\sigma\cdot k_{ex}=8\times 10^{-3}\,\tau_{B}^{-1}$ where $v_{ex}$ is the extrusion velocity. While these parameters were chosen to optimise simulation performances, the extruder density and the processivity ($\lambda=v_{ex}/k_{off}$ which in our case is $\lambda=160kbp$) are compatible with the ones found in literature (Fudenberg et al., 2016). The simulation type of each chromatin bead depends on the presence of epigenetic marks and DNA accessibility data for the genomic region covered by the bead. Three epigenetic marks are used: H3K27ac, H3K27me3 and H3K9me3 which are associated to transcriptionally active euchromatin, facultative heterochromatin and constitutive heterochromatin, respectively. Their ChIP-seq profiles for the HUVEC cell line are obtained from the ENCODE database. The presence of H3K27ac marks is used to place the additional crumpling springs, while H3K27me3 and H3K9me3 peaks determine the chromatin binding sites for polycomb-like and heterochromatin proteins, respectively. DNA accessibility is inferred from DHS data: DHS peaks in common between HUVEC and H1-hESC cells are used to know the position of active transcription factors which are cell generic, while DHS peaks present only in HUVEC cells determine the positions of HUVEC-specific active transcription factors. To include loop extrusion, CTCF binding sites have to be inserted in order to know where extruders halt. These are obtained from ChIP-seq peaks for CTCF which overlap with Rad21 ChIP-seq peaks. We then found the directionality of each site by locating its underlying binding motif. The center of the peak is mapped to a specific polymer bead that is so identified as a CTCF bead. To take into account cell-to-cell variability, in each repeated simulation we include only a part of CTCF beads, choosing them stochastically with a probability based on the peak height. All the data used as input are taken from the ENCODE database and are publicly available.
