# Supplementary material for: Cluster size determines internal structure of transcription factories in human cells
Source: eLife. 2026 Jul 20;14:RP103955. doi: 10.7554/eLife.103955 (PMC13384497; doi:10.7554/eLife.103955)
Supplement: Peer review history

# Editor report: eLife Assessment

**DOI:** 10.7554/eLife.103955.3.sa0

**Authors:** Timothy J Stasevich

**Affiliations:** Colorado State University United States

**Keywords:** Compelling, Valuable

This is a **valuable** polymer model that provides insight into the origin of macromolecular mixed and demixed states within transcription clusters. The simulations are well performed and clearly presented in the context of existing experimental datasets. This **compelling** study will be of interest to those studying gene expression in the context of chromatin.

# Referee report: Reviewer #1 (Public review)

**DOI:** 10.7554/eLife.103955.3.sa1

This manuscript discusses from a theory point of view he mechanisms underlying the formation of specialized or mixed factories. To investigate this, a chromatin polymer model was developed to mimic the chromatin binding-unbinding dynamics of various complexes of transcription factors (TFs).

The model revealed that both specialized (i.e., demixed) and mixed clusters can emerge spontaneously, with the type of cluster formed primarily determined by cluster size. Non-specific interactions between chromatin and proteins were identified as the main factor promoting mixing, with these interactions becoming increasingly significant as clusters grow larger.

These findings, observed in both simple polymer models and more realistic representations of human chromosomes, reconcile previously conflicting experimental results. Additionally, the introduction of different types of TFs was shown to strongly influence the emergence of transcriptional networks, offering a framework to study transcriptional changes resulting from gene editing or naturally occurring mutations.

Overall I think this is an interesting paper discussing a valuable model of how chromosome 3D organisation is linked to transcription.

Comments on revisions: It's a good paper.

# Referee report: Reviewer #2 (Public review)

**DOI:** 10.7554/eLife.103955.3.sa2

Summary:

With this report, I suggest what are in my opinion crucial additions to the otherwise very interesting and credible research manuscript "Cluster size determines morphology of transcription factories in human cells".

Strengths:

The manuscript in itself is technically sound, the chosen simulation methods are completely appropriate the figures are well-prepared, the text is mostly well-written spare a few typos. The conclusions are valid and would represent a valuable conceptual contribution to the field of clustering, 3D genome organization and gene regulation related to transcription factories, which continues to be an area of most active investigation.

Weaknesses:

However, I find that the connection to concrete biological data is weak. This holds especially given that the data that are needed to critically assess the applicability of the derived cross-over with factory size is, in fact, available for analysis, and the suggested experiments in the Discussion section are actually done and their results can be exploited. In my judgement, unless these additional analysis are added to a level that crucial predictions on TF demixing and transcriptional bursting upon TU clustering can be tested, the paper is more fitted for a theoretical biophysics venue than for a biology journal such as eLife.

Comments on revisions:

The authors have addressed my comments with exemplary diligence, which has clarified all my major concerns. In all cases, either the relevant work was added, or it was explained in the form of a convincing argument why the suggested modifications were not implemented or not possible to implement.

As a discretionary suggestion, the authors might consider using a title that even more directly highlights the, in my opinion, main take-away of this work. This is not because anything is incorrect about the current title, simply an even more to-the-point title might attract more readers. I would suggest something along the lines of

"Cluster size-dependent demixing drives specialization of transcription factories"

Overall, I congratulate the authors on their excellent work and appreciate the opportunity to engage with this manuscript during a very insightful review process.

# Referee report: Reviewer #3 (Public review)

**DOI:** 10.7554/eLife.103955.3.sa3

Summary:

In this work, the authors present a chromatin polymer model with some specific pattern of transcription units (TUs) and diffusing TFs; they simulate the model and study TFclustering, mixing, gene expression activity, and their correlations. First, the authors designed a toy polymer with colored beads of a random type, placed periodically (every 30 beads, or 90kb). These colored beads are considered a transcription unit (TU). Same-colored TUs attract with each other mediated by similarly colored diffusing beads considered as TFs. This led to clustering (condensation of beads) and correlated (or anti-correlation) "gene expression" patterns. Beyond the toy model, when authors introduce TUs in a specific pattern, it leads to emergence of specialized and mixed cluster of different TFs. Human chromatin models with realistic distribution of TUs also lead to the mixing of TFs when cluster size is large.

Strengths:

This is a valuable polymer model for chromatin with a specific pattern of TUs and diffusing TF-like beads. Simulation of the model tests many interesting ideas. The simulation study is convincing and the results provide solid evidence showing the emergence of mixed and demixed TF clusters within the assumptions of the model.

# Author comment: Author response

**DOI:** 10.7554/eLife.103955.3.sa4

**Authors:** Massimiliano Semeraro, Giuseppe Negro, Giada Forte, Antonio Suma, Giuseppe Gonnella, Peter Cook, Davide Marenduzzo

**Affiliations:** University of Bari Aldo Moro Bari Italy; University of Edinburgh Edinburgh United Kingdom; University of Edinburgh Edinburgh United Kingdom; Temple University Philadelphia United States; University of Bari Aldo Moro Bari Italy; University of Oxford Oxford United Kingdom; University of Edinburgh Edinburgh United Kingdom

The following is the authors’ response to the original reviews

**eLife Assessment**

This is a valuable polymer model that provides insight into the origin of macromolecular mixed and demixed states within transcription clusters. The well-performed and clearly presented simulations will be of interest to those studying gene expression in the context of chromatin. While the study is generally solid, it could benefit from a more direct comparison with existing experimental data sets as well as further discussion of the limits of the underlying model assumptions.

We thank the editors for their overall positive assessment. In response to the Referees’ comments, we have addressed all technical points, including a more detailed explanation of the methodology used to extract gene transcription from our simulations and its analogy with real gene transcription. Regarding the potential comparison with experimental data and our mixing–demixing transition, we have added new sections discussing the current state of the art in relevant experiments. We also clarify the present limitations that prevent direct comparisons, which we hope can be overcome with future experiments using the emerging techniques.

**Reviewer #1 (Public Review):**

This manuscript discusses from a theory point of view the mechanisms underlying the formation of specialized or mixed factories. To investigate this, a chromatin polymer model was developed to mimic the chromatin binding-unbinding dynamics of various complexes of transcription factors (TFs).

The model revealed that both specialized (i.e., demixed) and mixed clusters can emerge spontaneously, with the type of cluster formed primarily determined by cluster size. Non-specific interactions between chromatin and proteins were identified as the main factor promoting mixing, with these interactions becoming increasingly significant as clusters grow larger.

These findings, observed in both simple polymer models and more realistic representations of human chromosomes, reconcile previously conflicting experimental results. Additionally, the introduction of different types of TFs was shown to strongly influence the emergence of transcriptional networks, offering a framework to study transcriptional changes resulting from gene editing or naturally occurring mutations.

Overall I think this is an interesting paper discussing a valuable model of how chromosome 3D organisation is linked to transcription. I would only advise the authors to polish and shorten their text to better highlight their key findings and make it more accessible to the reader.

We thank the Referee for carefully reading our manuscript and recognizing its scientific value. As suggested, we tried to better highlight our key findings and make the text more accessible while addressing also the comments from the other Referees.

**Reviewer #2 (Public Review):**

Summary:

With this report, I suggest what are in my opinion crucial additions to the otherwise very interesting and credible research manuscript ”Cluster size determines morphology of transcription factories in human cells”.

Strengths:

The manuscript in itself is technically sound, the chosen simulation methods are completely appropriate the figures are well-prepared, the text is mostly well-written spare a few typos. The conclusions are valid and would represent a valuable conceptual contribution to the field of clustering, 3D genome organization and gene regulation related to transcription factories, which continues to be an area of most active investigation.

Weaknesses:

However, I find that the connection to concrete biological data is weak. This holds especially given that the data that are needed to critically assess the applicability of the derived cross-over with factory size is, in fact, available for analysis, and the suggested experiments in the Discussion section are actually done and their results can be exploited. In my judgement, unless these additional analysis are added to a level that crucial predictions on TF demixing and transcriptional bursting upon TU clustering can be tested, the paper is more fitted for a theoretical biophysics venue than for a biology journal such as eLife.

We thank the Reviewer for their positive assessment of the soundness of our work and its contribution to the field. We have added a paragraph to the Conclusions highlighting the current state of experimental techniques and outlining near-term experiments that could be extended to test our predictions. We also emphasise that our analysis builds on state-of-the-art polymer models of chromatin and on quantitative experimental datasets, which we used both to build the model construction and to validate its outcomes (gene activity). We hope this strengthened link to experiment will catalyse further studies in the field.

Major points:

(1) My first point concerns terminology.The Merriam-Webster dictionary describes morphology as the study of structure and form. In my understanding, none of the analyses carried out in this study actually address the form or spatial structuring of transcription factories. I see no aspects of shape, only size. Unless the authors want to assess actual shapes of clusters, I would recommend to instead talk about only their size/extent. The title is, by the same argument, in my opinion misleading as to the content of this study.

We agree with the Referee that the title could be misleading. In our study we characterized clusters size, that is a morphological descriptor, and cluster composition that isn’t morphology per se but used in the community in a broader sense. Nevertheless to strength the message we have changed the title in: “Cluster size determines internal structure of transcription factories in human cells”

(2) Another major conceptual point is the choice of how a single TF:pol particle in the model relates to actual macromolecules that undergo clustering in the cell. What about the fact that even single TF factories still contain numerous canonical transcription factors, many of which are also known to undergo phase separation? Mediator, CDK9, Pol II just to name a few. This alone already represents phase separation under the involvement of different species, which must undergo mixing. This is conceptually blurred with the concept of gene-specific transcription factors that are recruited into clusters/condensates due to sequencespecific or chromatin-epigenetic-specific affinities. Also, the fact that even in a canonical gene with a ”small” transcription factory there are numerous clustering factors takes even the smallest factories into a regime of several tens of clustering macromolecules. It is unclear to me how this reality of clustering and factory formation in the biological cell relates to the cross-over that occurs at approximately n=10 particles in the simulations presented in this paper.

This is a good point. However in our case we can either look at clustering transcription factors or transcription units. In an experimental situation, transcription units could be “coloured”, or assigned different types, by looking at different cell types, so that they can be classified as housekeeping, or cell-type independent, or cell-type specific. This is similar to how DHS can be clustered. In this way the mixing or demixing state can be identified by looking at the type of transcription unit, removing any ambiguity due to the fact that the same protein may participate in different TF complexes..

(3) The paper falls critically short in referencing and exploiting for analysis existing literature and published data both on 3D genome organization as well as the process of cluster formation in relation to genomic elements. In terms of relevant literature, most of the relevant body of work from the following areas has not been included:

(i) mechanisms of how the clustering of Pol II, canonical TFs, and specific TFs is aided by sequence elements and specific chromatin states

(ii) mechanisms of TF selectivity for specific condensates and target genomic elements

(iii) most crucially, existing highly relevant datasets that connect 3D multi-point contacts with transcription factor identity and transcriptional activity, which would allow the authors to directly test their hypotheses by analysis of existing data

Here, especially the data under point (iii) are essential. The SPRITE method (cited but not further exploited by the authors), even in its initial form of publication, would have offered a data set to critically test the mixing vs. demixing hypothesis put forward by the authors. Specifically, the SPRITE method offers ordered data on k-mers of associated genomic elements. These can be mapped against the main TFs that associate with these genomic elements, thereby giving an account of the mixed / demixed state of these k-mer associations. Even a simple analysis sorting these associations by the number of associated genomic elements might reveal a demixing transition with increasing association size k. However, a newer version of the SPRITE method already exists, which combines the k-mer association of genomic elements with the whole transcriptome assessment of RNAs associated with a particular DNA k-mer association. This can even directly test the hypotheses the authors put forward regarding cluster size, transcriptional activation, correlation between different transcription units’ activation etc.

To continue, the Genome Architecture Mapping (GAM) method from Ana Pombo’s group has also yielded data sets that connect the long-range contacts between gene-regulatory elements to the TF motifs involved in these motifs, and even provides ready-made analyses that assess how mixed or demixed the TF composition at different interaction hubs is. I do not see why this work and data set is not even acknowledged? I also strongly suggest to analyze, or if they are already sufficiently analyzed, discuss these data in the light of 3D interaction hub size (number of interacting elements) and TF motif composition of the involved genomic elements.

Further, a preprint from the Alistair Boettiger and Kevin Wang labs from May 2024 also provides direct, single-cell imaging data of all super-enhancers, combined with transcription detection, assessing even directly the role of number of super-enhancers in spatial proximity as a determinant of transcriptional state. This data set and findings should be discussed, not in vague terms but in detailed terms of what parts of the authors’ predictions match or do not match these data.

For these data sets, an analysis in terms of the authors’ key predictions must be carried out (unless the underlying papers already provide such final analysis results). In answering this comment, what matters to me is not that the authors follow my suggestions to the letter. Rather, I would want to see that the wealth of available biological data and knowledge that connects to their predictions is used to their full potential in terms of rejecting, confirming, refining, or putting into real biological context the model predictions made in this study.

References for point (iii):

- RNA promotes the formation of spatial compartments in the nucleus https://www.cell.com/cell/fulltext/S0092-8674(21)01230-7?dgcid=raven_jbs_etoc_email

- Complex multi-enhancer contacts captured by genome architecture mapping https://www.nature.com/articles/nature21411

- Cell-type specialization is encoded by specific chromatin topologies https://www.nature.com/articles/s41586-021-04081-2

- Super-enhancer interactomes from single cells link clustering and transcription https://www.biorxiv.org/content/10.1101/2024.05.08.593251v1.full

For point (i) and point (ii), the authors should go through the relevant literature on Pol II and TF clustering, how this connects to genomic features that support the cluster formation, and also the recent literature on TF specificity. On the last point, TF specificity, especially the groups of Ben Sabari and Mustafa Mirx have presented astonishing results, that seem highly relevant to the Discussion of this manuscript.

We appreciate the Reviewer’s insightful suggestion that a comparison between our simulation results and experimental data would strengthen the robustness of our model. In response, we have thoroughly revised the literature on multi-way chromatin contacts, with particular attention to SPRITE and GAM techniques. However, we found that the currently available experimental datasets lack sufficient statistical power to provide a definitive test of our simulation predictions, as detailed below.

As noted by the Reviewer, SPRITE experiments offer valuable information on the composition of highorder chromatin clusters (k-mers) that involve multiple genomic loci. A closer examination of the SPRITE data (e.g., Supplementary Material from Ref. [1]) reveals that the majority of reported statistics correspond to 3-mers (three-way contacts), while data on larger clusters (e.g., 8-mers, 9-mers, or greater) are sparse. This limitation hinders our ability to test the demixing-mixing transition predicted in our simulations, which occurs for cluster sizes exceeding 10.

Moreover, the composition of the k-mers identified by SPRITE predominantly involves genomic regions encoding functional RNAs—such as ITS1 and ITS2 (involved in rRNA synthesis) and U3 (encoding small nucleolar RNA)—which largely correspond to housekeeping genes. Conversely, there is little to no data available for protein-coding genes. This restricts direct comparison to our simulations, where the demixing-mixing transition depends critically on the interplay between housekeeping and tissue-specific genes.

Similarly, while GAM experiments are capable of detecting multi-way chromatin contacts, the currently available datasets primarily report three-way interactions [2,3].

In summary, due to the limited statistical data on higher-order chromatin clusters [4], a quantitative comparison between our simulation results and experimental observations is not currently feasible. Nevertheless, we have now briefly discussed the experimental techniques for detecting multi-way interactions in the revised manuscript to reflect the current state of the field, mentioning most of the references that the Reviewer suggested.

(4) Another conceptual point that is a critical omission is the clarification that there are, in fact, known large vs. small transcription factories, or transcriptional clusters, which are specific to stem cells and ”stressed cells”. This distinction was initially established by Ibrahim Cisse’s lab (Science 2018) in mouse Embryonic Stem Cells, and also is seen in two other cases in differentiated cells in response to serum stimulus and in early embryonic development:

- Mediator and RNA polymerase II clusters associate in transcription-dependent condensates https://www.science.org/doi/10.1126/science.aar4199

- Nuclear actin regulates inducible transcription by enhancing RNA polymerase II clustering https://www.science.org/doi/10.1126/sciadv.aay6515

- RNA polymerase II clusters form in line with surface condensation on regulatory chromatin https://www.embopress.org/doi/full/10.15252/msb.202110272

- If ”morphology” should indeed be discussed, the last paper is a good starting point, especially in combination with this additional paper: Chromatin expansion microscopy reveals nanoscale organization of transcription and chromatin https://www.science.org/doi/10.1126/science.ade5308

We thank the Reviewer for pointing out the discussion about small and large clusters observed in stressed cells. Our study aims to provide a broader mechanistic explanation on the formation of TF mixed and demixed clusters depending on their size. However, to avoid to generate confusion between our terminology and the classification that is already used for transcription factories in stem and stressed cells, we have now added some comments and references in the revised text.

(5) The statement scripts are available upon request is insufficient by current FAIR standards and seems to be non-compliant with eLife requirements. At a minimum, all, and I mean all, scripts that are needed to produce the simulation outcomes and figures in the paper, must be deposited as a publicly accessible Supplement with the article. Better would be if they would be structured and sufficiently documented and then deposited in external repositories that are appropriate for the sharing of such program code and models.

We fully agree with the Reviewer. We have now included in the main text a link to an external repository containing all the codes required to reproduce and analyze the simulations.

**Recommendations for the authors:**

Minor and technical points

(6) Red, green, and yellow (mix of green and red) is a particularly bad choice of color code, seeing that red-green blindness is the most common color blindness. I recommend to change the color code.

We appreciate the Reviewer’s thoughtful comment regarding color accessibility. We fully agree that red–green combinations can pose challenges for color-blind readers. In our figures, however, we chose the red–green–yellow color scheme deliberately because it provides strong contrast and intuitive representation for different TF/TU types. To ensure accessibility, we optimized brightness and saturation within red-green schemes and we carefully verified that the chosen hues are distinguishable under the most common forms of color vision deficiency, i.e. trichromatic color blindness, using color-blindness simulation tools (e.g., Coblis).

How is the dispersing effect of transcriptional activation and ongoing transcription accounted for or expected to affect the model outcome? This affects both transcriptional clusters (they tend to disintegrate upon transcriptional activation) as well as the large scale organization, where dispersal by transcription is also known.

We thank the Reviewer for this very insightful question. The current versions of both our toy model and the more complex HiP-HoP model do not incorporate the effects of RNA Polymerase elongation. Our primary goal was to develop a minimalisitc framework that focuses on investigating TF clusters formation and their composition. Nevertheless, we find that this straightforward approach provides a good agreement between simulations and Hi-C and GRO-seq experiments, lending confidence to the reliability of our results concerning TF cluster composition.

We fully agree, however, that the effects of transcription elongation are an interesting topic for further exploration. For example, modeling RNA Polymerases as active motors that continually drive the system out of equilibrium could influence the chromatin polymer conformation and the structure of TF clusters. Additionally, investigating how interactions between RNA molecules and nuclear proteins, such as SAF-A, might lead to significant changes in 3D chromatin organization and, consequently, transcription [5], is also in intriguing prospect. Although we do not believe that the main findings of our study, particularly regarding cluster composition and mixed-demixed transition, would be impacted by transcription elongation effects, we recognize the importance of this aspect. As such, we have now included some comments in the Conclusions section of the revised manuscript.

“and make the reasonable assumption that a TU bead is transcribed if it lies within 2.25 diameters (2.25σ) of a complex of the same colour; then, the transcriptional activity of each TU is given by the fraction of time that the TU and a TF:pol lie close together.” How is that justified? I do not see how this is reasonable or not, if you make that statement you must back it up.

As pointed out by the Referee, we consider a TU to be active if at least one TF is within a distance 2.25σ from that TU. This threshold is a slightly larger than the TU-TF interaction cutoff distance, *r_c_* \ = 1.8σ between TFs and TUs. The rationale for this choice is to ensure that, in the presence of a TU cluster surrounded by TFs, TUs that are not directly in contact with a TF are still considered active. Nonetheless, we find that using slightly different thresholds, such as 1.8σ or 1.1σ, leads to comparable results, as shown in Fig. S11, demonstrating the robustness of our analysis.

Clearly, close proximity in 1D genomic space favours formation of similarly-coloured clusters. This is not surprising, it is what you built the model to do. Should not be presented as a new insight, but rather as a check that the model does what is expected.

We believed that this sentence already conveyed that the formation of single-color clusters driven by 1D genomic proximity is not a surprising outcome. However, we have now slightly rephrased it to better emphasize that this is not a novel insight.

That said, we would like to highlight that while 1D genomic proximity facilitates the formation of clusters of the same color, the unmixed-to-mixed transition in cluster composition is not easily predictable solely from the TU color pattern. Furthermore, in simulations of real chromosomes, where TU patterns are dictated by epigenetic marks, the complexity of these patterns makes it challenging—if not impossible—to predict cluster composition based solely on the input data of our model.

“…how closely transcriptional activities of different TUs correlate…” Please briefly state over what variable the correlation is carried out, is it cross correlation of transcription activity time courses over time? Would be nice to state here directly in the main text to make it easier for the reader.

We have now included a brief description in the revised manuscript explaining how the transcriptional correlations were evaluated and how the correlation matrix was constructed.

“The second concerns how expression quantitative trait loci (eQTLs) work. Current models see them doing so post-transcriptionally in highly-convoluted ways [11, 55], but we have argued that any TU can act as an eQTL directly at the transcriptional level [11].” This text does not actually explain what eQTLs do. I think it should, in concise words.

We agree with the Referee’s suggestion. We have revised the sentence accordingly and now provide a clear explanation of eQTLs upon their first mention. The revised paragraph now reads as follows:

“The second concerns how expression quantitative trait loci (eQTLs)—genomic regions that are statistically associated with variation in gene expression levels—function. While current models often attribute their effects to post-transcriptional regulation through complex mechanisms [6,7], we have previously argued that any transcriptional unit (TU) can act as an eQTL by directly influencing gene expression at the transcriptional level [7]. Here, we observe individual TUs up-regulating or down-regulating the activity of others TUs – hallmark behaviors of eQTLs that can give rise to genetic effects such as “transgressive segregation” [8]. This phenomenon refers to cases in which alleles exhibit significantly higher or lower expression of a target gene, and can be, for instance, caused by the creation of a non-parental allele with a specific combination of QTLs with opposing effects on the target gene.”

“In the string with 4 mutations, a yellow cluster is never seen; instead, different red clusters appear and disappear (Fig. 2Eii)…” How should it be seen? You mutated away most of the yellow beads. I think the kymograph is more informative about the general model dynamics, not the effects of mutations. Might be more appropriate to place a kymograph in Figure 1.

We agree with the Referee that the kymograph is the most appropriate graphical representation for capturing the effects of mutations. Panel 2E already refers to the standard case shown in Figure 1. We have now clarified this both in the caption and in the main text. In addition, we have rephrased the sentence—which was indeed misleading—as follows:

“From the activity profiles in Fig. 2C, we can observe that as the number of mutations increases, the yellow cluster is replaced by a red cluster, with the remaining yellow TUs in the region being expelled (Fig. 2B(ii)). This behavior is reflected in the dynamics, as seen by comparing panels E(i) and E(ii): in the string with four mutations, transcription of the yellow TUs is inhibited in the affected region, while prominent red stripes—corresponding to active, transcribing clusters—emerge (Fig. 2E(ii)).” We hope that the comparison is now immediately clear to the reader.

“…but this block fragments in the string with 4 mutations…” I don’t know or cannot see what is meant by ”fragmentation” in the correlation matrix.

With the sentence “this block fragments in the string with 4 mutations” we mean that the majority of the solid red pixels within the black box become light-red or white once the mutations are applied. We have now added a clarification of this point in the revised manuscript.

“Fig. 3D shows the difference in correlation between the case with reduced yellow TFs and the case displayed in Fig. 1E.” Can you just place two halves of the different matrices to be compared into the same panel? Similar to Fig. S5. Will be much easier to compare.

We thank the Referee for this suggestion. We tried to implement this modification, and report the modified figure below (Author response image 1). As we can see, in the new figure it is difficult to spot the details we refer to in the main text, therefore we prefer to keep the original version of the figure.

**Author response image 1.** Heatmap comparing activity correlations of TUs in the random string under normal conditions (top half) and with reduced yellow-TF concentration (bottom half).

What is the omnigenic model? It is not introduced.

We thank the Reviewer for highlighting this important point. The omnigenic model, first introduced by Boyle et al in Ref. [6], was proposed to explain how complex traits, including disease risk, are influenced by a vast number of genes. Accordingly to this model, the genetic basis of a trait is not limited to a small set of core genes whose expression is directly related to the trait, but also includes peripheral genes. The latter, although not directly involved in controlling the trait, can influence the expression of core genes through gene regulatory networks, thereby contributing to the overall genetic influence on the trait. We have now added a few lines in the revised manuscript to explain this point.

“Additionally, blue off-diagonal blocks indicate repeating negative correlations that reflect the period of the 6-pattern.” How does that look in a kymograph? Does this mean the 6 clusters of same color steal the TFs from the other clusters when they form?

The intuition of the Referee is indeed correct. The finite number of TFs leads to competition among TUs of the same colour, resulting in anticorrelation:when a group of six nearby TUs of a given colour is active, other, more distant TUs of the same colour are not transcribing due to the lack of available TFs. As the Referee suggested,this phenomenon is visible in the kymograph showing TU activity. In Author response image 2, it can be observed that typically there is a single TU cluster for each of the three colours (yellow, green, and red). These clusters can be long-lived (e.g., the yellow cluster at the center of the kymograph) or may destroy during the simulation (e.g., the red cluster at the top of the kymograph, which dissolves at *t* ∼ 600 × 10^5^ *τ_B_*). In the latter case, TFs of the corresponding colour are released into the system and can bind to a different location, forming a new cluster (as seen with the red cluster forming at the bottom of the kymograph for *t >* 600 × 10^5^ *τ_B_*). This point is further discussed at the point 2.30 of this Reply where additional graphical material is provided.

**Author response image 2.** Kymograph showing the TU activity during a typical run in the 6-pattern case. Each row reports the transcriptional state of a TU during one simulation. Black pixels correspond to inactive TUs, red (yellow, green) pixels correspond to active red (yellow, green) TUs.

“Conversely, negative correlations connect distant TUs, as found in the single-color model…” But at the most distal range, the negative correlation is lost again! Why leave this out? Your correlation curves show the same , equilibration towards no correlation at very long ranges.

As highlighted in Figure 5Ai, long-range negative correlations (grey segments) predominantly connect distant TUs of the same colour. This is quantified in Figure 5Bi: restricting to same-colour TUs shows that at large genomic separations the correlation is almost entirely negative, with small fluctuations at distances just below 3000 kbp where sampling is sparse; we therefore avoid further interpretation of this regime.

“These results illustrate how the sequence of TUs on a string can strikingly affect formation of mixed clusters; they also provide an explanation of why activities of human TUs within genomic regions of hundreds of kbp are positively correlated [60].” This is a very nice insight.

We thank the Reviewer for the very supportive comment.

“To quantify the extent to which TFs of different colours share clusters, we introduce a demixing coefficient, *θ_dem_* (defined in Fig. 1).” This is not defined in Fig. 1 or anywhere else here in the main text.

We thank the Referee for pointing this out. For a given cluster, the demixing coefficient is defined as

$\theta_{\mathrm{dem}}=\frac{n x_{i, \max }-1}{n-1},$

where *n* is the number of colors, *i* indexes each color present in the model, and *x_i,max_* the largest fraction of TFs of the same *i*-th color in a single TF cluster.

The demixing coefficient is defined in the Methods section; therefore, we have replaced defined in Fig. 1 with see Methods for definition.

“Mixing is facilitated by the presence of weakly-binding beads, as replacing them with non-interacting ones increases demixing and reduces long-range negative correlations (Figure S3). Therefore, the sequence of strong and weak binding sites along strings determines the degree of mixing, and the types of small-world network that emerge. If eQTLs also act transcriptionally in the way we suggest [11], we predict that down-regulating eQTLs will lie further away from their targets than up-regulating ones.” Going into these side topics and minke points here is super distracting and waters down the message. Maybe first deal with the main conclusions on mixed vs demixed clusters in dependence on the strong and specific binding site patterns, before dealing with other additional points like the role of weak binding sites.

Thank you for the suggestion. We now changed the paragraph to highlight the main results. The new paragraph is as follows. “These results on activity correlation and TF cluster composition suggest that, if eQTLs act transcriptionally as expected [7], down-regulating eQTLs are likely to be located further from their target genes than up-regulating ones. In addition, it is important to note that mixing is promoted by the presence of weakly binding beads; replacing these with non-interacting ones leads to increased demixing and a reduction in long-range negative correlations (Figure S3). More generally, our findings indicate that the presence of multiple TF colors offers an effective mechanism to enrich and fine-tune transcriptional regulation.”

“…provides a powerful pathway to enrich and modulate transcriptional regulation.” Before going into the possible meaning and implications of the results, please discuss the results themselves first.

See previous point.

Figure 5B. Does activation typically coincide with spatial compaction of the binding sites into a small space or within the confines of a condensate? My guess would be that colocalization of the other color in a small space is what leads to the mixing effect?

As the Reviewer correctly noted, the activity of a given TU is indeed influenced by the presence of nearby TUs of the same color, since their proximity facilitates the recruitment of additional TFs and enhances the overall transcriptional activity. In this context, the mixing effect is certainly affected by the 1D arrangement of TUs along the chromatin fiber. As emphasized in the revised manuscript, when domains of same-color TUs are present (as in the 6-pattern string), the degree of demixing is greater compared to the case where TUs of different colors alternate and large domains are absent (as in the 1-pattern string). This difference in the demixing parameter as a function of the 1D TU arrangement is clearly visible in Fig. S2B.

“…euchromatic regions blue, and heterochromatic ones grey.” Please also explain what these color monomers mean in terms of non specific interactions with the TFs.

Generally, in our simulation approach we assume euchromatin regions to be more open and accessible to transcription factors, whereas heterochromatin corresponds to more compacted chromatin segments [9]. To reflect this, we introduce weak, non-specific interactions between euchromatin and TFs, while heterochromatin interacts with TFs only thorugh steric effects. To clarify this point, we have now slightly revised the caption of Fig.6.

“More quantitatively, Spearman’s rank correlation coefficient is 3.66 10^−1^, which compares with 3.24 10^−1^ obtained previously using a single-colour model [11].” This comparison does not tell me whether the improvement in model performance justifies an additional model component. There are other, likelihood based approaches to assess whether a model fits better in a relevant extent by adding a free model parameter. Can these be used for a more conclusive comparison? Besides, a correlation of 0.36 does not seem so good?

We understand the Reviewer’s concern that the observed increase in the activity correlation may not appear to provide strong evidence for the improvement of the newly introduced model. However, within the context of polymer models developed to study realistic gene transcription and chromatin organization, this type of correlation analysis is a widely accepted approach for model validation. Experimental data commonly used for such validation include Hi-C maps, FISH experiments, and GRO-seq data [10,11]. The first two are typically employed to assess how accurately the model reproduces the 3D folding of chromatin; a comparison between experimental and simulated Hi-C maps is provided in the Supplementary Information (Fig. S5), showing a Pearson correlation of 0.7. GRO-seq or RNA-seq data, on the other hand, are used to evaluate the model’s ability to predict gene transcription levels. To date, the highest correlation for transcriptional activity data has been achieved by the HiP-HoP model at a resolution of 1 kbp [10], reporting a Spearman correlation of 0.6. Therefore, the correlation obtained with our 2-color model represents a good level of agreement when compared with the more complex HiP-HoP model. In this context, the observed increase in correlation—from 0.324 to 0.366—can be regarded as a modest yet meaningful improvement.

“…consequently, use of an additional color provides a statisticallysignificant improvement (p-value < 10^−6^, 2-sided t-test).” I do not follow this argument. Given enough simulation repeats, any improvement, no matter how small, will lead to statistically significant improvements.

We agree that this sentence could be misleading. We have now rephrased it in a clearer manner specifying that each of the two correlation values is statistically significant alone, while before we were wrongly referring to the significance of the improvement.

“Additionally, simulated contact maps show a fair agreement with Hi-C data (Figure S5), with a Pearson correlation r ∼ 0.7 (p-value < 10^−6^, 2-sided t-test).” Nice!

We thank the Reviewer for the positive comment.

“Because we do not include heterochromatin-binding proteins, we should not however expect a very accurate reproduction of Hi-C maps: we stress that here instead we are interested in active chromatin, transcription and structure only as far as it is linked to transcription.” Then why do you not limit your correlation assessment to only these regions to show that these are very well captured by your model?

We thank the Reviewer for this insightful comment. Indeed, we could have restricted our investigation to active chromatin regions, as done in our previous works [11,12]. However, our intention in this section of the manuscript was to clarify that the current model is relatively simple and therefore not expected to achieve a very high level of agreement between experimental and simulated Hi-C maps. Another important limitation of the two color model described in the section is the absence of active loop extrusion mediated by SMC proteins, which is known to play a central role in establishing TADs boundaries. Consequently, even if our analysis were limited to active chromatin regions, the agreement with experimental Hi-C maps would still remain lower than that obtained with more comprehensive models, such as HiP-HoP, that we use later in the last section of the paper. We have now added a comment in the revised manuscript explicitly noting the lack of active loop extrusion in our 2-color model.

“We also measure the average value of the demixing coefficient, *θ_dem_* (Materials and Methods). If *θ_dem_* = 1, this means that a cluster contains only TFs of one colour and so is fully demixed; if *θ_dem_* = 0, the cluster contains a mixture of TFs of all colors in equal number, and so is maximally mixed.” Repetitive.

We have now rephrased the sentence in a more concise way.

“…notably, this is similar to the average number of productivelytranscribing pols seen experimentally in a transcription factory [6].” That seems a bit fast and loose. The number of Polymerases can differ depending on state, type of factory, gene etc. and vary between anything from to a few hundreds of Polymerase complexes depending on definition of factory, and what is counted as active. Also, one would think that polymerases only make up a small part of the overall protein pool that constitutes a condensate, so it is unclear whether this is a pertinent estimate.

Here we refer to the average size of what is normally referred to as a PolII factory, not a generic nuclear condensate. These are the clusters which arise in our simulations. These structures emerge through microphase separation and have been well characterised, for instance see [13] for a recent review. For these structures while there is a distribution the average is well defined and corresponds to a size of about 100 nm, which is very much in line with the size of the clusters we observe, both in terms of 3D diameter and number of participating proteins. Because of the size, the number of active complexes which can contribute cannot be significantly more than ∼ 10. These estimates are, we note, very much in line with super-resolution measurements of SAF-A clusters [14], which are associated with active transcription and hence it is reasonable to assume they colocalise with RNA and polymerase clusters.

“Conversely, activities of similar TUs lying far from each other on the genetic map are often weakly negatively correlated, as the formation of one cluster sequesters some TFs to reduce the number available to bind elsewhere.” This point is interesting, and I strongly suspect that this indeed happening. But I don’t think it was shown in the analysis of the simulation results in sufficient clarity. We need direct assessment of this sequestration, currently it’s only indirectly inferred.

Indeed, this is the mechanism underlying the emergence of negative long-range correlations among TU activity values. As the Reviewer correctly pointed out, the competition for a finite number of TFs was only indirectly inferred in the original manuscript. To address this, we have now included a new figure explicitly illustrating this effect. In Fig. S12, we show the kymograph of active TUs (left panel), as in Fig. 2E(i) of the main text, alongside a new kymograph depicting the number of green TFs within a sphere of radius 10σ centered on each green TU (right panel). For simplicity, we focus here only on green TUs and TFs. It can be observed that, during the initial part of the simulation, green TFs are localized near genomic position ∼ 2000(right panel), where green TUs are transcriptionally active (left panel). Toward the end of the simulation, TUs near genomic position ∼ 500 become active, coinciding with the relocation of TFs to this region and the depletion of the previous one.

In the definition for the demixing coefficient (equation 1), what does the index i stand for?

Here *i* is an index denoting each of the colors present in the model. We have now specified the meaning of *i* after Eq. 1.

**Reviewer 3 (Public Review):**

In this work, the authors present a chromatin polymer model with some specific pattern of transcription units (TUs) and diffusing TFs; they simulate the model and study TFclustering, mixing, gene expression activity, and their correlations. First, the authors designed a toy polymer with colored beads of a random type, placed periodically (every 30 beads, or 90kb). These colored beads are considered a transcription unit (TU). Same-colored TUs attract with each other mediated by similarly colored diffusing beads considered as TFs. This led to clustering (condensation of beads) and correlated (or anti-correlation) ”gene expression” patterns. Beyond the toy model, when authors introduce TUs in a specific pattern, it leads to emergence of specialized and mixed cluster of different TFs. Human chromatin models with realistic distribution of TUs also lead to the mixing of TFs when cluster size is large.

Strengths.

This is a valuable polymer model for chromatin with a specific pattern of TUs and diffusing TF-like beads. Simulation of the model tests many interesting ideas. The simulation study is convincing and the results provide solid evidence showing the emergence of mixed and demixed TF clusters within the assumptions of the model.

Weaknesses.

Weakness of the work: The model has many assumptions. Some of the assumptions are a bit too simplistic. Concerns about the work are detailed below:

We thank the Referee for this overall positive evaluation.

We thank the Referee for this important observation. The way we The authors assume that when the diffusing beads (TFs) are near a TU, the gene expression starts. However, mammalian gene expression requires activation by enhancer-promoter looping and other related events. It is not a simple diffusion-limited event. Since many of the conclusions are derived from expression activity, will the results be affected by the lack of looping details?

We do not need to assume promoter-enhancer contact, this emerges naturally through the bridging-induced phase separation and indeed is a key strength of our model. Even though looping is not assumed as key to transcriptional initiation, in practice the vast majority of events in which a TF is near a TU are associated with the presence of a cluster where regulatory elements are looped. So transcription in our case is associated with the bridging-induced phase separation, and there is no lack of looping, looping is naturally associated with transcription, and this is an emergent property of the model (not an assumption), which is an important feature of our model. Accordingly, both contact maps and transcriptional activity are well predicted by our model, both in the version described here and in the more sophisticated single-colour HiP-HoP model [10] (an important ingredient of which is the bridging-induced phase separation).

Authors neglect protein-protein interactions. Without proteinprotein interactions, condensate formation in natural systems is unlikely to happen.

We thank the Reviewer for pointing out the absence of protein-protein interactions in our simulations. While we acknowledge this limitation, we would like to emphasize that experimental studies have not observed nuclear proteins forming condensates at physiological concentrations in the absence of DNA or chromatin. For example, studies such as Ryu et al. [15] and Shakya et al. [16] show that protein-protein interactions alone are insufficient to drive condensate formation in vivo. Instead, the presence of a substrate, such as DNA or chromatin, is essential to favor and stabilize the formation of protein clusters.

In our simulations, we propose that protein liquid-liquid phase separation (LLPS) is driven by the presence of both strong and weak attractions between multivalent protein complexes and the chromatin filament. As stated in our manuscript, the mechanism leading to protein cluster formation is the bridging induced attraction. This mechanism involves a positive feedback loop, where protein binding to chromatin induces a local increase in chromatin density, which then attracts more proteins, further promoting cluster formation.

While we acknowledge that adding protein-protein interactions could be incorporated into our simulations, we believe this would need to be a weak interaction to remain consistent with experimental data. Additionally, incorporating such interactions would not alter the conclusions of our study.

What is described in this paper is a generic phenomenon; many kinds of multivalent chromatin-binding proteins can form condensates/clusters as described here. For example, if we replace different color TUs with different histone modifications and different TFs with Hp1, PRC1/2, etc, the results would remain the same, wouldn’t they? What is specific about transcription factor or transcription here in this model? What is the logic of considering 3kb chromatin as having a size of 30 nm? See Kadam et al. (Nature Communications 2023). Also, DNA paint experimental measurement of 5kb chromatin is greater than 100 nm (see work by Boettiger et al.).

We thank the Reviewer for this important observation, which we now address. To begin, we consider the toy model introduced in the first part of the manuscript, where TUs are randomly positioned rather than derived from epigenetic data. As the Reviewer points out, in this simplified context, our results reflect a generic phenomenon: the composition of clusters depends primarily on their size, independent of the specific types of proteins involved. However, the main goal of our work is to gain insights into apparently contradictory experimental findings, which show that some transcription factories consist of a single type of transcription factors, while other contain multiple types. This led us to focus on TF clusters and their role in transcriptional regulation and co-regulation of distant genes. Therefore, in the second part of the manuscript, we use DNase I hypersensitive site (DHS) data to position TUs based on predicted TF binding sites, providing a more biological framework. In both the toy model and the more realistic HiP-HoP model, we observe a size-dependent transition in cluster composition. However, we refrain from generalizing these results to clusters composed of other protein complexes, such as HP1 and PRC, as their binding is governed by distinct epigenetic marks (e.g. *H3K927me3* and *H3K27me3*), which exhibit different genomic distributions compared to DHS marks.

Finally, the mapping of 3kb to 30nm is an estimate which does not significantly impact our conclusions. The relationship between genomic distance (in kbp) and spatial distance (in nm) is highly dependent on the degree of chromatin compaction, which can vary across cell types and genomic context. As such, providing an exact conversion is challenging [17]. For example, in a previous work based on the HiP-HoP model [12] we compared simulated and experimental FISH measurements and found that 1kbp typically corresponds to 15 − 20nm, implying that 3kbp could span 60nm. Nevertheless, we emphasize that varying this conversion factor does not affect the core results or conclusions of our study. We have now included a clarification in the revised SI to highlight this point.

**Recommendations for the authors:**

Other points.

Figure 1(D) caption says 2.25σ = 1.6 nanometer. Is this a typo? Sigma is 30nm.

Yes, it was. As 1σ ∼ 30nm, we have 2.25σ = 2.25 · 30 nm = 67.2 nm ∼ 6.7 × 10^−8^m. We have now corrected the caption.

Page 6, column 2nd, 3rd para, it is written that *θ_dem_* (”defined in Fig.1”). There is no *θ_dem_* defined in Fig.1, is there? I can see it defined in Methods but not in Fig. 1.

Correct, we replaced (defined in Fig.1) with (see Methods for definition).

Page 6, column 2, 4th para: what does “correlations overlap and correlations diverge mean”?

With reference to the plots from Fig. 5B, correlation overlap and diverge simply refers to the fact that same-colour (red curves) and different-colour (blue curves) correlation trends may or may not overlap on each other. We have now clarified this point.

What is the precise definition of correlation in Fig 5B (Y-axis)?

In Fig.5B, correlation means Pearson correlation. We have now specified this point in the revised text and in the caption of Fig.5.

References

(1) S. A. Quinodoz, J. W. Jachowicz, P. Bhat, N. Ollikainen, A. K. Banerjee, I. N. Goronzy, M. R. Blanco, P. Chovanec, A. Chow, Y. Markaki et al., “Rna promotes the formation of spatial compartments in the nucleus,” Cell, vol. 184, no. 23, pp. 5775–5790, 2021.

(2) R. A. Beagrie, A. Scialdone, M. Schueler, D. C. Kraemer, M. Chotalia, S. Q. Xie, M. Barbieri, I. de Santiago, L.-M. Lavitas, M. R. Branco et al., “Complex multi-enhancer contacts captured by genome architecture mapping,” Nature, vol. 543, no. 7646, pp. 519–524, 2017.

(3) R. A. Beagrie, C. J. Thieme, C. Annunziatella, C. Baugher, Y. Zhang, M. Schueler, A. Kukalev, R. Kempfer, A. M. Chiariello, S. Bianco et al., “Multiplex-gam: genome-wide identification of chromatin contacts yields insights overlooked by hi-c,” Nature Methods, vol. 20, no. 7, pp. 1037–1047, 2023.

(4) L. Liu, B. Zhang, and C. Hyeon, “Extracting multi-way chromatin contacts from hi-c data,” PLOS Computational Biology, vol. 17, no. 12, p. e1009669, 2021.

(5) R.-S. Nozawa, L. Boteva, D. C. Soares, C. Naughton, A. R. Dun, A. Buckle, B. Ramsahoye, P. C. Bruton, R. S. Saleeb, M. Arnedo et al., “Saf-a regulates interphase chromosome structure through oligomerization with chromatin-associated rnas,” Cell, vol. 169, no. 7, pp. 1214–1227, 2017.

(6) E. A. Boyle, Y. I. Li, and J. K. Pritchard, “An expanded view of complex traits: from polygenic to omnigenic,” Cell, vol. 169, no. 7, pp. 1177–1186, 2017.

(7) C. Brackley, N. Gilbert, D. Michieletto, A. Papantonis, M. Pereira, P. Cook, and D. Marenduzzo, “Complex small-world regulatory networks emerge from the 3d organisation of the human genome,” Nat. Commun., vol. 12, no. 1, pp. 1–14, 2021.

(8) R. B. Brem and L. Kruglyak, “The landscape of genetic complexity across 5,700 gene expression traits in yeast,” Proceedings of the National Academy of Sciences, vol. 102, no. 5, pp. 1572– 1577, 2005.

(9) M. Chiang, C. A. Brackley, D. Marenduzzo, and N. Gilbert, “Predicting genome organisation and function with mechanistic modelling,” Trends in Genetics, vol. 38, no. 4, pp. 364–378, 2022.

(10) M. Chiang, C. A. Brackley, C. Naughton, R.-S. Nozawa, C. Battaglia, D. Marenduzzo, and N. Gilbert, “Genome-wide chromosome architecture prediction reveals biophysical principles underlying gene structure,” Cell Genomics, vol. 4, no. 12, 2024.

(11) A. Buckle, C. A. Brackley, S. Boyle, D. Marenduzzo, and N. Gilbert, “Polymer simulations of heteromorphic chromatin predict the 3d folding of complex genomic loci,” Mol. Cell, vol. 72, no. 4, pp. 786–797, 2018.

(12) G. Forte, A. Buckle, S. Boyle, D. Marenduzzo, N. Gilbert, and C. A. Brackley, “Transcription modulates chromatin dynamics and locus configuration sampling,” Nature Structural & Molecular Biology, vol. 30, no. 9, pp. 1275–1285, 2023.

(13) P. R. Cook and D. Marenduzzo, “Transcription-driven genome organization: a model for chromosome structure and the regulation of gene expression tested through simulations,” Nucleic acids research, vol. 46, no. 19, pp. 9895–9906, 2018.

(14) M. Marenda, D. Michieletto, R. Czapiewski, J. Stocks, S. M. Winterbourne, J. Miles, O. C. Flemming, E. Lazarova, M. Chiang, S. Aitken et al., “Nuclear rna forms an interconnected network of transcription-dependent and tunable microgels,” BioRxiv, pp. 2024–06, 2024.

(15) J.-K. Ryu, C. Bouchoux, H. W. Liu, E. Kim, M. Minamino, R. de Groot, A. J. Katan, A. Bonato, D. Marenduzzo, D. Michieletto et al., “Bridging-induced phase separation induced by cohesin smc protein complexes,” Science advances, vol. 7, no. 7, p. eabe5905, 2021.

(16) A. Shakya, S. Park, N. Rana, and J. T. King, “Liquid-liquid phase separation of histone proteins in cells: role in chromatin organization,” Biophysical journal, vol. 118, no. 3, pp. 753–764, 2020.

(17) A.-M. Florescu, P. Therizols, and A. Rosa, “Large scale chromosome folding is stable against local changes in chromatin structure,” PLoS computational biology, vol. 12, no. 6, p. e1004987, 2016.
